# Lewis acid protection turns cyanide containing [FeFe]-hydrogenase mimics into proton reduction catalysts†

**DOI:** 10.1039/d1dt03896f

**Published:** 2022-02-18

**Authors:** Holly J. Redman, Ping Huang, Michael Haumann, Mun Hon Cheah, Gustav Berggren

**Affiliations:** Department of Chemistry – Ångström Laboratory, Uppsala University Box 523 75120 Uppsala Sweden gustav.berggren@kemi.uu.se; Department of Physics, Freie Universität Berlin Arnimallee 14 14195 Berlin Germany

## Abstract

Sustainable sources of hydrogen are a vital component of the envisioned energy transition. Understanding and mimicking the [FeFe]-hydrogenase provides a route to achieving this goal. In this study we re-visit a molecular mimic of the hydrogenase, the propyl dithiolate bridged complex [Fe_2_(μ-pdt)(CO)_4_(CN)_2_]^2−^, in which the cyanide ligands are tuned *via* Lewis acid interactions. This system provides a rare example of a cyanide containing [FeFe]-hydrogenase mimic capable of catalytic proton reduction, as demonstrated by cyclic voltammetry. EPR, FTIR, UV-vis and X-ray absorption spectroscopy are employed to characterize the species produced by protonation, and reduction or oxidation of the complex. The results reveal that biologically relevant iron-oxidation states can be generated, potentially including short-lived mixed valent Fe(i)Fe(ii) species. We propose that catalysis is initiated by protonation of the diiron complex and the resulting di-ferrous bridging hydride species can subsequently follow two different pathways to promote H_2_ gas formation depending on the applied reduction potential.

## Abstract 2

Mimicking the hydrogen-bonding interactions of the [FeFe]-hydrogenase active-site using Lewis acids transforms an otherwise unstable cyanide containing hydrogenase mimic into a proton reduction catalyst.

## Graphical abstract

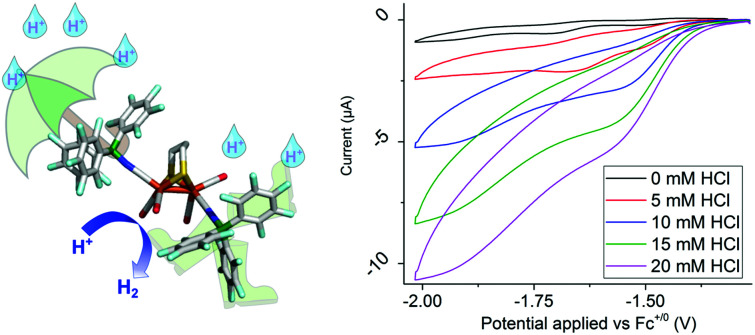

## Introduction

**Fig. 1:**
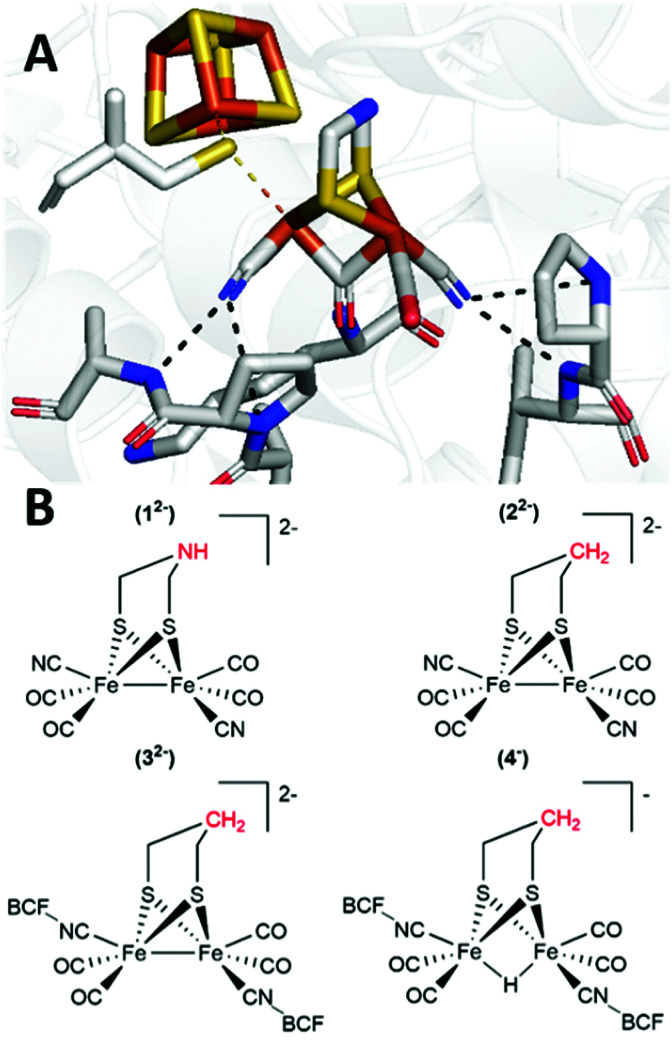
Panel A: The H-cluster, the active site of [FeFe]-hydrogenase, consisting of a [4Fe–4S] cluster fused with the dinuclear [2Fe]_H_ subsite (adapted from entry 6SG2 in the Protein Databank).^9–11^ The black dashed lines represent hydrogen bonding of the cyanide ligands of the [2Fe]_H_ subsite, to amino acids of the protein (P108 and A109; I204 and P203); and the yellow dashes denote linking of the [4Fe–4S]_H_ and [2Fe]_H_ sub-complexes by a bridging cystein thiol. Panel B: Cyanide substituted structural mimics of the [2Fe]_H_ subsite with different central groups in the bridging dithiolate ligand, [Fe_2_(μ-adt)(CO)_4_(CN)_2_]^2−^ (1^2−^) and [Fe_2_(μ-pdt)(CO)_4_(CN)_2_]^2−^ (2^2−^); [Fe_2_(μ-pdt)(CO)_4_(CN-BCF)_2_]^2−^ (3^2−^) is formed upon BCF addition to 2^2−^; and [(μ-H)Fe_2_(μ-pdt)(CO)_4_(CN-BCF)_2_]^−^ (4^−^) is a bridging hydride species formed by protonation of 3^2−^.

[FeFe]-hydrogenases are a structurally and functionally diverse family of enzymes, with the most efficient examples reported to-date reducing protons to dihydrogen (H_2_) with turnover frequencies (TOF) of up to 10 000 s^−1^.^1^ Iron is one of the most abundant elements in the Earth's crust, making the [FeFe]-hydrogenase a promising system to study for renewable hydrogen production as an alternative to platinum driven electrolysis.^2^ All [FeFe]-hydrogenases feature the same hexanuclear iron active-site, known as the H-cluster (Fig. 1A).^3–5^ It consists of a typical [4Fe–4S] cluster, coupled to an organometallic diiron cofactor *via* a bridging cysteine thiol ([2Fe]_H_). The biologically unique [2Fe]_H_-cofactor is the site of catalysis; and its iron centers are low valent, presumably cycling between [Fe(i)Fe(i)] and [Fe(ii)Fe(ii)] during catalysis. They share a bridging azadithiolate ligand (–SCH_2_NHCH_2_S–, adt), and are further coordinated by the strong field ligands CN^−^ and CO, stabilizing a low spin state.^6–9^

Thus, the aforementioned bridging cysteine thiol is the only covalent bond that anchors the [2Fe]_H_ subsite in the active site pocket of the enzyme.^6^ However, additional stabilization is provided by surrounding amino acids interacting with the cyanide ligands *via* hydrogen bonds (Fig. 1A), while the carbonyl ligands are positioned in hydrophobic pockets.^9^

Hydrogen production evidently involves proton coupled electron transfer, and terminal hydride species are generally considered as key intermediates in the enzyme. However, the role of bridging hydrides during catalytic turnover, or regulation processes, is debated.^12–15^ In addition to metal hydrides, the nitrogen bridgehead of the cofactor and the [4Fe4S] cluster have been proposed as protonation sites.^3^

[FeFe]-hydrogenases have promising prospects for biotechnological applications. Unravelling the enzyme's mechanism has also guided the design of improved synthetic catalysts for H^+^/H_2_ interconversion. Consequently, a wealth of H-cluster, or more specifically, [2Fe]_H_ subsite mimics has been reported. Indeed, to-date over 1000 diiron-carbonyl complexes have been listed in the Cambridge Crystallographic Database.^4,16,17^ Such mimics are not only explored for their catalytic properties, but also serve as model systems for understanding fundamental aspects of the H-cluster. Arguably, one of the closest structural molecular mimics of the [FeFe]-cofactor is the complex [Fe_2_(μ-adt)(CO)_4_(CN)_2_]^2−^ (1^2−^, Fig. 1B), reported already in 2002.^18,19^ It has been shown that 1^2−^ is unstable in solution under acidic conditions,^20,21^ but when inserted into apo-[FeFe]-hydrogenase it generates a semi-synthetic hydrogenase as active as the native enzyme.^8^ This finding highlights the importance of an outer coordination sphere for the function of 1^2−^ and related mimics. A closely related well characterized structural mimic is the analogous propane dithiolate bridged complex [Fe_2_(μ-pdt)(CO)_4_(CN)_2_]^2−^ (2^2−^, Fig. 1B).^20,22,23^

Such hydrogenase mimics are generally studied by FTIR, EPR, NMR, UV-Vis spectroscopy, and their electrocatalytic activity is commonly addressed by electrochemical techniques.^24,25^ The carbonyl and cyanide ligands exhibit strong vibrational bands in a characteristic region of the infrared spectrum, thus FTIR spectroscopy is an ideal complement to EPR for studying [FeFe]-hydrogenases.^3^ Further details about oxidation state and structure of the iron centers can be obtained by X-ray absorption spectroscopy (XAS).^26,27^

In this study, we investigate a previously reported [2Fe]_H_ mimic on which the cyanides of 2^2−^ are capped with the bulky Lewis acid, tris(pentafluorophenyl)borane (BCF) (3^2−^, Fig. 1B).^28^ Complex 2^2−^ with the carbon (–pdt–) bridgehead was selected as it allows probing of the diiron core and its related iron hydride(s) without interference from protonation at the nitrogen of the adt bridgehead (1^2−^). As reported by Manor *et al.* the borane caps the cyanide ligands, ensuring that they are protected from decomposition by protonation, and also enabled formation of the corresponding bridging hydride complex (4^−^).^28^ In a broader context, the addition of Lewis acids has been reported to enable tuning of the electron density and catalytic properties of various cyanide and nitrile containing metal complexes.^28–32^ Specifically in a hydrogenase context this interaction mimics the hydrogen bonding of the protein to the cyanide ligands of the [2Fe]_H_ subsite (Fig. 1A) and enables the study of the catalytic activity of structurally related dicyanide mimics, which has previously been challenging.^33–35^

Here we show that the binding of BCF to complex 2^2−^ turns it into an electrocatalyst for H_2_ production. The change in Fe electron density following protonation of the Fe(i)Fe(i) dimer to form the di-ferrous hydride is probed by X-ray and FTIR spectroscopy. Through a combination of spectroscopy and cyclic voltammetry we propose that the catalytic cycle includes Fe(i)Fe(i), Fe(i)Fe(ii) and Fe(ii)Fe(ii) intermediates. These results for the dicyanide complex 2^2−^ highlight the strong influence of the outer coordination sphere on this class of complexes. More specifically, it underscores the importance of hydrogen bonding to the cyanide ligands in the active-site pocket. Albeit the proposed catalytic mechanism of the mimic proceeds *via* bridging hydride species, the biologically relevant oxidation states implicated in the suggested catalytic cycle(s) provide a strong biomimetic aspect.

## Experimental section

### General

Chemicals were purchased from Merck/Sigma Aldrich and used as received unless otherwise noted. Solvents were purified on an InertSolv solvent purification system and stored in an MBraun LabStar glovebox kept under argon atmosphere at <0.5 ppm H_2_O and O_2_ for up to 1 month prior to usage. The quality of employed iron-carbonyl complexes were verified by FTIR before use. FTIR absorption spectra were recorded on solution samples between 2250 and 1600 cm^−1^ on a Bruker (Vertex 70v) spectrometer using a liquid nitrogen cooled MCT (mercury cadmium telluride) detector controlled with OPUS software (spectral resolution 2 cm^−1^). The IR measurements were performed with a demountable FTIR liquid cell (Pike Technologies) using CaF_2_ windows with 0.2 mm PTFE spacers.

X-band EPR measurements were performed on a Bruker ELEXYS E500 spectrometer equipped with a SuperX EPR049 microwave bridge and a cylindrical TE_011_ ER 4122SHQE cavity equipped with a continuous flow cryostat (Oxford Instruments), and using an ITC 503 temperature controller (Oxford Instruments). The Xepr software package (Bruker) was used for data acquisition and processing of spectra. The EasySpin software (version easyspin-6.0.0-dev.34) was used for spectral simulation and fitting.^36,37^ Measurement temperatures ranged from 10 to 40 K, using liquid helium as the coolant. The following EPR settings were used unless otherwise stated: microwave power 1 mW, modulation amplitude 1 mT, modulation frequency 100 kHz.

UV-Vis spectra were collected using gas tight quartz cells with 1 cm optical path lengths, using a Varian Cary 100 Bio UV–vis spectrophotometer.

### Preparation of compounds

#### Synthesis of Fe_2_(μ-pdt)(CO)_6_ and [Fe_2_(μ-pdt)(CO)_4_(CN)_2_]^2−^

Preparation of Fe_2_(μ-pdt)(CO)_6_, and 2^2−^ were done by literature procedures.^20,38^

#### Synthesis of Fe_2_(μ-pdt)(CO)_4_(CN-BCF)_2_ (3^2−^)

Synthesis of 3^2−^ was carried out by literature procedures with minor modifications.^28^ In the glovebox, a dry Schlenk flask was charged with 2^2−^ (0.922 g, 1.43 mmol), and tris(pentafluorophenyl)borane (1.455 g, 2.85 mmol). The Schlenk flask was transferred from the glovebox to the Schlenk line, and degassed. The flask was backfilled with argon and then charged with dry degassed dichloromethane (20 cm^3^) *via* canula. The reaction mixture was stirred at room temperature for 30 min, followed by evaporation of solvent under reduced pressure to afford a red-brown solid. The product was suspended and stirred in pentane for 3 hours to clean and subsequently filtered and dried under vacuum. This yielded a red-orange microcrystalline solid. Yield: 1.5 g, 76%. IR (CH_2_Cl_2_) *ṽ*/cm^−1^ = 2136, 1990, 1954, 1922.

#### Synthesis of [(μ-H)Fe_2_(μ-pdt)(CO)_4_(CN-BCF)_2_] (4^−^)

In the glovebox, 4^−^ was prepared in solution by charging a scintillation vial with 3^2−^ (16.67 mg, 10 μmol) and dissolving in MeCN (2 cm^3^) to make a 5 mM solution of 3^2−^. To this solution HCl (40 μL 0.125 M) was added that had previously been prepared by dilution of 1 M HCl in Et_2_O into MeCN. Addition of HCl resulted in a slight colour change of the solution, from orange to pale orange. The resulting product was observed by IR (CH_3_CN) *ṽ*/cm^−1^ = 2186, 2070, 2050, 2020.

### Chemical redox titrations

#### Chemical oxidation

A solution of 3^2−^ was prepared (5 mM, 2 mL) and aliquoted into 250 μL portions. A solution of AgNO_3_ (E *vs.* Fc^+/0^ = 0.04 V, 5 mM, 1 mL) was prepared. The AgNO_3_ solution was titrated into each aliquot of 3^2−^ as follows; 25 μL (0.1 eq.); 100 μL (0.4 eq.); 125 μL (0.5 eq.); 150 μL (0.6 eq.); 200 μL (0.8 eq.); 250 μL (1.0 eq.). Each titration point was monitored by FTIR (ESI_10 and 11†), the end point was recorded by UV-vis and EPR spectroscopy. End point IR (MeCN) *ṽ*/cm^−1^ = 2151, 2009, 1989, 1953; UV-Vis *λ*_max_/nm = 346.

#### Chemical reduction

**Fig. 2:**
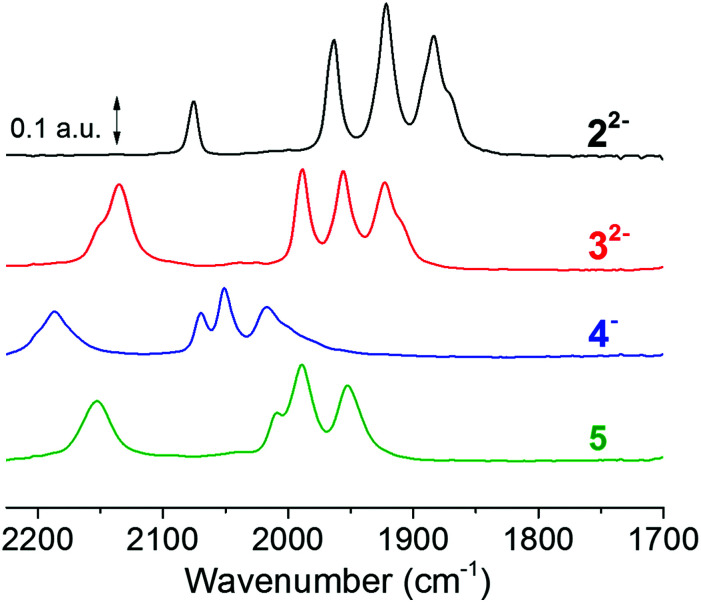
FTIR spectra of studied complexes, 2^2−^ (black spectrum), its corresponding borane adduct 3^2−^ (red spectrum), and the protonated borane adduct 4^−^ (blue spectrum). Spectra were recorded on 0.5 mM solutions of the complexes in acetonitrile; 4^−^ was prepared by adding 4 eq. of HCl (2 mM) to a 0.5 mM solution of 3^2−^; and 3^2−^ was treated with AgNO_3_ at room temperature to give 5 (green spectrum) IR band frequencies are summarised in Table 1 (corresponding EPR spectra are shown in Fig. S17†).

A solution of 4^−^ was prepared as described above (5 mM, 5 ml) and aliquoted into 500 μL portions. Another solution of decamethylcobaltocene (CoCp*) was prepared separately (E *vs.* Fc^+/0^ = −1.94 V, 50 mM, 1 ml). This solution was titrated into the solution of 4^−^ as follows; 50 μL (1 eq.); 100 μL (2 eq.); 150 μL (3 eq.); 200 μL (4 eq.). The end point was recorded by FTIR, EPR and UV-vis spectroscopy (Fig. 2 and ESI_2 Fig. S4, ESI_11 Fig. S16†). End point IR (MeCN) *ṽ*/cm^−1^ = 2135, 1988, 1955, 1922; UV-Vis *λ*_max_/nm = 346.

### X-ray absorption spectroscopy

X-ray absorption spectroscopy (XAS) samples were prepared at Uppsala University. A solution of 3^2−^ (500 μL, 5 mM in MeCN) was prepared and mixed with HCl (50 μL 0.2 M), or AgNO_3_ (12.5 μL 0.2 M in MeCN) to make 4^−^ or 5, to provide a total iron concentration of ∼10 mM in the samples. Mixing was followed by injection into Kapton covered Delrin holders (100 μL). After the samples were loaded into the sample holders, they were removed from the glovebox and immediately frozen in an isopropanol-N_2_(l) bath before transfer to liquid nitrogen. The XAS measurements at the Fe K-edge were performed at beamline KMC-3 at the BESSY-II synchrotron (Helmholtz Center Berlin, Germany; 250 mA top-up mode of the storage ring) as described earlier,^39^ using a set-up including a Si[111] double-crystal monochromator, a 13-element energy-resolving Si-drift detector (RaySpec), and DXP-XMAP pulse-processing electronics (XIA). Samples were held at 20 K in a closed-cycle liquid-helium cryostat (Oxford). The energy axis of the monochromator was calibrated (accuracy ± 0.1 eV) using the K-edge spectrum of an iron metal foil (fitted reference energy of 7112 eV in the first derivative spectrum). The spot size on the samples was *ca.* 2.0 × 4.0 mm (vertical × horizontal) as set by a focusing mirror and slits. X-ray fluorescence spectra were collected using a continuous monochromator-scan mode (scan duration ∼15 min). Up to 6 scans to *k* = 16.2 Å^−1^ were averaged (2 scans per sample spot) for improving the signal-to-noise ratio. XAS data were processed (dead-time correction, background subtraction, normalization) to yield XANES and EXAFS spectra using our earlier described procedures and in-house software.^26,40–42^*k*^3^-Weighted EXAFS spectra were simulated with in-house software and phase functions from FEFF9 (*S*_0_^2^ = 0.8).^43^

### Cyclic voltammetry

Cyclic voltamograms (CVs) were recorded using a 1 mm diameter glassy carbon working electrode, a titanium counter electrode with a 2.5 μm thick platinum coating, and a leakless miniature Ag|AgCl reference electrode, all purchased from eDAQ. A 3 mL scintillation vial was used as an electrochemical cell, with a custom made Teflon lid with holes for the electrodes. Ferrocene was added routinely at the end of the experiment as an internal reference, and measured potentials were aligned to the ferrocene/ferrocenium (Fc^+/0^) reference. The glassy carbon working electrode was polished using 0.3 μm alumina powder slurry in distilled water, followed by 0.05 μm alumina powder slurry in distilled water, and then sonicated in EtOH for 10 min, and dried before use. The working electrode was pre-treated by scanning at 250 mV s^−1^ from −2 to +1.7 V in 0.2 M [NBu_4_][PF_6_]. The open circuit potential (OCP) was determined before recording the CVs, and the start and end-points were at the measured OCP. Data analysis was carried out assuming a planar working electrode with surface area of 0.00785 cm^2^.

Cyclic voltammetry traces were obtained using a 5 mM solution of 3^2−^ in a scan velocity range of 0.005 V s^−1^ to 5 V s^−1^. Where indicated, hydrochloric acid (HCl, 1 M in diethylether) was titrated into the electrochemical cell to generate compound 4^−^*in situ*, as demonstrated by FTIR results. CVs were subsequently recorded at 100 mV s^−1^ in the potential ranges −2.01–1.25 V, and −1.0–0 V *vs.* Fc^+/0^ (for further details see ESI_5–8†).

## Results and discussion

### Preparation and characterization of 2^2−^, 3^2−^, 4^−^

The borane capped Fe(i)Fe(i) complex, 3^2−^, was obtained as an orange microcrystalline powder by treating the dicyanide complex 2^2−^ with two eqs of the Lewis acid, BCF. The change in colour of the solution from burgundy-red 2^2−^ to orange-red 3^2−^ was accompanied by a 30 cm^−1^ hypsochromic shift in the carbonyl bands (Fig. 2). This was expected from the decreased electron density on the Fe-ions and in good agreement with the earlier report of Manor *et al.*^28^ The observed shift is significantly larger than the shift of ∼10 cm^−1^ previously reported following protonation of the cyanide ligands, indicating a relatively stronger electron withdrawing effect of the BCF.^33,44^ Furthermore, the cyanide band was shifted by 60 cm^−1^ to higher frequencies, and increased in relative intensity. This larger shift of the cyanide bands is reflective of the strong influence of BCF on the cyanide ligands *via* direct through-bond interactions. The protonation of 3^2−^ to produce the (formally) diferrous complex 4^−^ was previously described in dichloromethane. In order to enable subsequent XAS studies (which are difficult to perform in CH_2_Cl_2_ due to the strong X-ray absorbance of this solvent) *vide infra*, we explored the chemistry of 3^2−^ and 4^−^ in acetonitrile. Treatment of 3^2−^ (500 μL, 5 mM) with HCl (4 μL, 1 M) resulted in a visible change in the appearance of the solution from orange-red to pale orange. Compound 3^2−^ (0.5 mM) showed an absorption at 346 nm (abs = 1.98, *ε*_3FeFe_ = 3960 L mol^−1^ cm^−1^) which is assigned to an MMCT transition involving the Fe–Fe bond.^45^ There was an additional absorption at 503 nm (abs = 0.14, *ε*_3ππ_ = 289 L mol^−1^ cm^−1^) that was assigned to pi-pi transition of the phenyl rings of the BCF moieties.

Titration of HCl into a solution of 3^2−^ to form 4^2−^ was monitored by UV-Vis spectroscopy. The absorption at 503 nm changed very little between titration points, while the absorption at 346 nm was significantly diminished at larger HCl concentrations. A loss of the latter band upon protonation of the Fe–Fe bond has previously been reported for related di-phosphine complexes,^47^ and can be attributed to the predicted transition from a diiron metal–metal bond to a three-center-two-electron bond attributed to the Fe-(μ-H)–Fe moiety.^48,49^ New features become visible at 326 nm (Abs = 0.62, *ε*_326 nm_ = 1240 L mol^−1^ cm^−1^) and 382 nm (Abs = 0.41, *ε*_382 nm_ = 820 L mol^−1^ cm^−1^) (ESI_2, Fig. S3†).

Oxidative addition of a proton to the Fe–Fe bond is confirmed by FTIR spectroscopy. When a solution of 3^2−^ in dry acetonitrile was treated with four eqs of HCl, a spectral shift to higher frequencies was observed. The carbonyl bands were shifted by approximately 90 cm^−1^, while the cyanide band shifted by 50 cm^−1^. In combination, these observations demonstrate that the Lewis acid protection and protonation chemistry previously reported in dichloromethane is reproducible in acetonitrile.

As bridging hydrides have been proposed to be present in the so-called H_red_H^+^ and H_sred_H^+^ states of the H-cluster (also referred to as H_red_ and H_sred_, respectively, in some reports), we utilized this biomimetic complex to search for H/D isotope effects on the positions of the carbonyl bands. Additional FTIR studies were carried out in which DCl was added to compound 3^2−^. This resulted in an identical FTIR spectrum as observed when 3^2−^ was treated with HCl (ESI_3, Fig. S5 and Table S2†). Thus, in contrast to terminal hydrides,^50^ bridging hydrides are unlikely to be easily inferable from H/D exchange and analysis of CO/CN IR-band positions. Notably, unaltered CO/CN band positions were observed also for the above-mentioned H-cluster species for H/D exchange.^14^ Finally, to explore the possibility of transient terminal hydride formation on route to the final bridging hydride species, protonation with HCl was studied by stopped-flow rapid-scan FTIR spectroscopy. The rate constant for the binuclear reaction was determined to be *k*_1_ ∼ 16 ± 6 L mol^−1^ s^−1^, with no indication of any intermediate species (ESI_4, Fig. S7 and Table S3†).

### Generation of mixed valent Fe(i)Fe(ii) species

Fe(i)Fe(ii) species are central to the mechanism of the native H-cluster, and are observed in both the H_red_ and H_ox_ states.^3,4^ Corresponding model complexes have primarily been prepared using phosphine ligands.^51,52^ The instability of cyanide ligated diiron complexes has thus far prevented the investigation of these closer structural analogues in such mixed valent oxidation states.^20,33^

#### Chemical oxidation of 3^2−^

Treatment of 3^2−^ with AgNO_3_ as an oxidant (*E*°(Ag|Ag^+^) = 0.04 V *vs.* Fc^+/0^)^53^ resulted in the solution becoming visually darker. The UV-vis spectrum showed that the feature at 346 nm, broadens (ESI_9, Fig. S14†). The oxidation of 3^2−^ was also readily observable by FTIR spectroscopy, as titration with AgNO_3_ resulted in a new species with a clearly distinct FTIR spectrum (Fig. 2 and ESI_10, Fig. S15†). Overall, the carbonyl bands display a hypsochromic shift of approximately 40 cm^−1^, as compared to the original FTIR spectrum of 3^2−^. Three carbonyl and one cyanide bands are still discernible, but the high frequency carbonyl bands at 2009 cm^−1^ and 1989 cm^−1^ begin to coalesce. Complete conversion of 3^2−^ was achieved with 1 eq. of AgNO_3_ (relative to 3^2−^). This is in agreement with a one-electron oxidation to yield a mixed valent Fe(i)Fe(ii) state, analogous to H_ox_ of the enzyme's catalytic cycle. However, more complicated reactivity is indicated by a decrease in absorbance of the carbonyl bands by about 50%. Indeed, EPR spectra recorded on samples collected after oxidation of 3^2−^ by AgNO_3_ at room temperature reveal that the product is EPR silent (ESI_12, Fig. S17†), which does not support a mixed valent state as expected from a clean one-electron oxidation of 3^2−^ to yield 3^−^. Therefore, if formed, any Fe(i)Fe(ii) intermediate must quickly react further to form an EPR silent species. Obtaining FTIR spectra of samples prepared under low temperature conditions was not achievable with our available instrumentation. Thus, to further probe the oxidation of 3^2−^ by AgNO_3_, EPR samples were prepared by oxidizing 3^2−^ at −70 °C. The resulting EPR spectrum showed a weak, but clearly visible, isotropic signal at *g* = 2.022 (Fig. S17†).

Temperature and power studies suggest that the EPR signal is attributable to a paramagnetic metal complex and not to a (*e.g.* ligand) radical. Incubating the EPR sample for two min at room temperature resulted in a complete loss of the signal (Fig. S17†).

Based on the combination of FTIR and EPR data, we attribute the hypsochromically shifted FTIR spectrum to an EPR silent complex, denoted as 5. The isotropic EPR signal obtained from mixing at low temperature is instead tentatively attributed to the mixed valent complex 3^−^. However, the available data does not allow us to fully rule out that the signal arises from a short-lived degradation product. If formed, 3^−^ is evidently highly unstable in MeCN and we propose that this complex rapidly forms 5. Upon addition of NaBH_4_ or CoCp* to freshly made solutions of 5, approximately 30% of the initial concentration of 3^2−^ is recovered as determined by FTIR spectroscopy (ESI_11, Fig. S16†). This demonstrates that although the oxidation is electrochemically irreversible (*vide infra*), it is partially chemically reversible on a min time-scale. This strongly suggests that all four carbonyl ligands of 3^2−^ are retained in 5. Still, the loss of a significant amount of the original signal is an indicator that the transition of 3^2−^ into 5 involves partial degradation to give a species not readily discernible by FTIR or EPR spectroscopy. The exact structure of 5 remains to be elucidated, but some information was obtained from XAS (*vide infra*). There is precedence in the literature for dimerization of related diiron complexes, but the bulky borane capping ligands makes this unlikely. Another option is comproportionation of the Fe(i)Fe(ii) complex.^38,54,55^

However, it should be noted that the EPR inactive nature of 5 is unlikely to be attributable to a two electron oxidation, to yield a di-ferrous species, as the hypsochromic of the carbonyl bands is relatively modest and only one equivalent of oxidant is needed for complete conversion of 3^2−^ to 5 (ESI_10, Fig. S15†). Further investigation of the oxidation of 3^2−^ and reduction of 5 is needed to fully disentangle this chemistry.

#### Chemical reduction of 4^−^

**Fig. 3:**
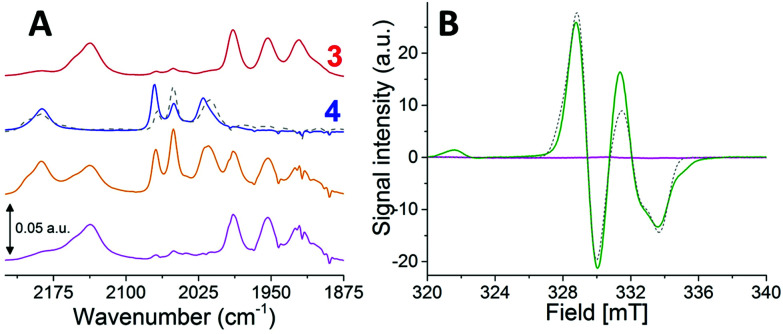
The reduction of 4^−^ followed by FTIR (panel A) and EPR (panel B) spectroscopy. Panel A: FTIR spectra following the addition of CoCp* to complex 4^−^. 5 mM 3^2−^ (red spectrum); 5 mM 4^−^ (blue spectrum); 5 mM 4^−^ + 20 mM (4 eq.) CoCp* collected 3 min after mixing (orange spectrum), revealing a mixture of 3^2−^ and 4^−^; 5 mM 4^−^ + 20 mM (4 eq.) CoCp* collected 10 min after mixing (magenta spectrum), revealing complete conversion to 3^2−^. The contributions of a possible intermediate at the 3 min time-point is shown as a grey dashed line. Panel B: EPR spectrum following the addition of CoCp* to 4^−^ at −40 °C (green spectrum), showing a mixture of two paramagnetic species; simulated EPR spectrum following addition of CoCp* to 4^−^ (grey dashed line, for details see Fig. S20†) and spectrum recorded following incubation at room temperature for 5 min (magenta spectrum), yielding an EPR silent product.

When 4 eqs of the one-electron reductant CoCp* is added to 4^−^ in the presence of protons, complex 3^2−^ is re-formed within 10 min as observed by FTIR and UV/Vis spectroscopy (Fig. 3A and ESI_2, Fig. S4†). More specifically, 66% of 3^2−^ is recovered, based on the reappearance of the peak in the UV-vis spectrum at 346 nm. Close examination of the relative peak intensities in the spectrum recorded 3 min after addition of CoCp* to 4^−^ (Fig. 3A, orange spectrum) hints at the presence of an intermediate, which we were not able to isolate in room temperature FTIR experiments. A difference spectrum in which contributions from complex 3^2−^ (magenta spectrum) and 4^−^ (blue spectrum) have been subtracted from the orange spectrum is shown in Fig. 3A (grey dashed line).

EPR spectroscopy was employed to monitor the reduction reaction at decreased temperatures, analogously to the oxidation chemistry. Samples collected from solutions of 4^−^ reduced by mixing with CoCp* at −40 °C revealed EPR spectra reflecting at least two distinct paramagnetic species. One rhombic species *g*_1,2,3_ = 2.039, 2.015 and 2.004 and one narrow axial species *g*_⊥_ = 2.033, *g*_∥_ = 2.027 in a ratio of 3 : 1 rhombic : axial (ESI_13, Fig. S20†). Temperature studies demonstrated that both components of the spectrum decreased significantly in intensity as temperature was increased from 10 K to 40 K (ESI_13, Fig. S18†). Conversely, neither the rhombic nor the axial component showed strong saturation tendencies within the studied microwave power range (0.1 to 10 mW), even at the lowest measured temperature (ESI_13, Fig. S19†). X-band EPR spectroscopy alone does not allow for a complete structural elucidation, but these observations again suggest that the EPR signals are attributable to mixed valent metal species rather than radicals. The presence of two different paramagnetic species upon reduction is potentially due to partial degradation or differences in protonation state. However, considering the low temperature nature of the experiment and the fact that earlier NMR studies have shown that 4^−^ adopts two major isomeric hydrides,^28^ it is more likely attributable to the structural isomerism of 4^−^. Thus, we propose that the two EPR signals observed in the samples generated at −40 °C is the result of reduction of these two isomers, as previously reported for the mixed-valent hydride complex (μ-H)Fe_2_(pdt)(CO)_2_(dppv)_2_ (dppv = *cis*-1,2-C_2_H_2_(PPh_2_)_2_).^56^ The absence of a distinct hyperfine coupling pattern due to the hydrogen nuclear spin would in this case reflect limited coupling to the hydride ligand.^35^

Subsequent incubation of the sample for 5 min at room temperature resulted in a complete loss of the EPR signals (Fig. 3B, magenta spectrum). The diamagnetic nature of the product obtained at room temperature is in good agreement with the proposed formation of 3^2−^ based on FTIR.

**Scheme 1:**
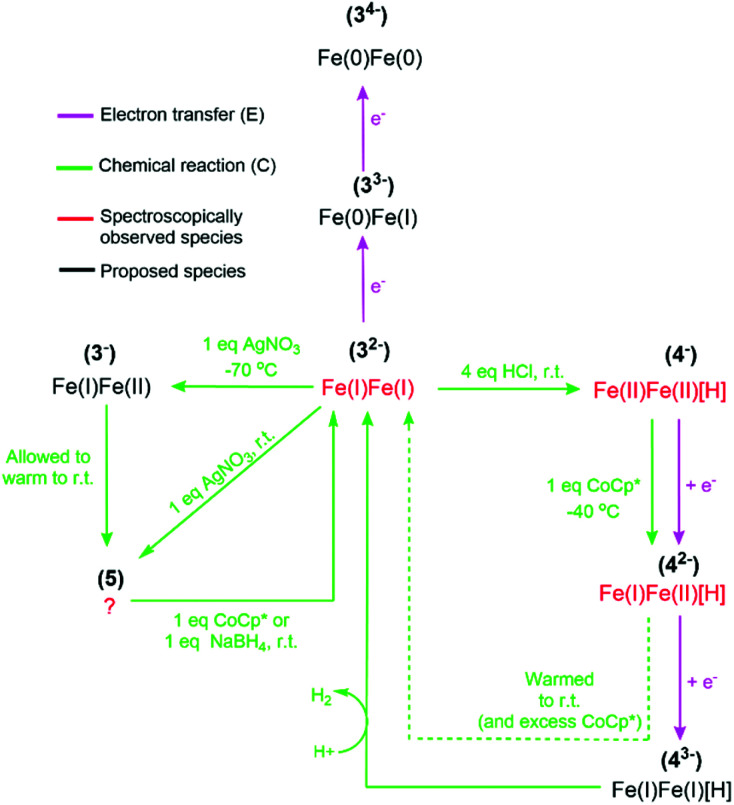
Schematic overview summarising the observed redox and protonation chemistry of 3^2−^. Note that 4^2−^ is proposed based on X-band EPR. The chemical reagents employed to trigger a specific reaction are shown, while an electrochemical redox process is indicated by “e^−^”.

As summarized in Scheme 1, these results support the notion that mixed valent Fe(i)Fe(ii) species can be formed from the reduction of 4^−^, and potentially also from the oxidation of 3^2−^. However, both 3^−^ and 4^2−^ are unstable at room temperature and rapidly convert to 5 and 3^2−^, respectively. The observation that 4^−^ regenerates 3^2−^ under reducing conditions indicates that the complex is capable of catalytic proton reduction. The catalytic properties of 3^2−^ and its related hydride species were further analyzed by electrochemistry, *vide infra*.

### X-ray absorption spectroscopy

**Fig. 4:**
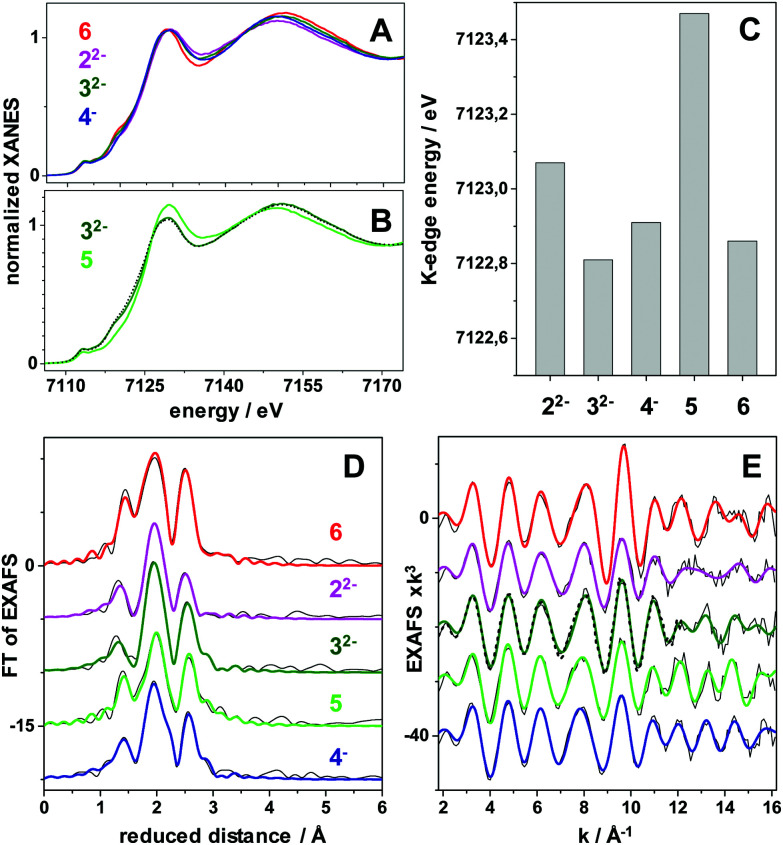
X-ray absorption spectroscopy data at the Fe K-edge of diiron complexes in MeCN solution. (A and B) X-ray absorption near edge structure (XANES) spectra of indicated complexes. (C) Fe K-edge energies (at 50% level) of the XANES spectra. (D) Fourier-transforms of the extended X-ray absorption fine structure (EXAFS) spectra in (E) of the complexes (black lines, experimental data; coloured lines, simulations with parameters in Table S4†). The annotations refer to the complexes shown in Fig. 1. The spectrum denoted 5 is the result of oxidizing 3^2−^ with AgNO_3_ (5 is also observed by UV-vis and IR spectroscopy in Fig. S13 and S14†). 6 is Fe_2_(μ-pdt)(CO)_6_. The black dashed lines in (B and E) show the spectra of 4^−^ after reduction with CoCp to regain 3^2−^.

X-ray absorption spectroscopy (XAS) was employed to obtain more detailed insight into the oxidation states and solution structures of the iron complexes. We obtained XAS spectra of complexes 3^2−^ and 4^−^ in solution, as well as 3^2−^ after AgNO_3_ addition (*i.e.*5) (Fig. 4A–C and ESI_17, Fig. S26, 27†). In addition, XAS data for the reference compounds Fe_2_(μ-pdt)(CO)_6_ (6) and 2^2−^ were collected. The spectra (meaning the almost unchanged shape of the XANES and the metrical parameters from EXAFS analysis) show that the molecular integrity of the compounds remained intact in solution after hydride formation (4^−^) and oxidation (5). Furthermore, the EXAFS analysis shows that, in those cases where X-ray crystallographic data is available (2^2−^, 3^2−^, and 6), the solution structures are in good agreement with the available crystal structures (Fig. 4D and E and ESI_17, Fig. S26, S27†).

Moreover, all complexes display relatively subtle differences in Fe K-edge energies and Fe–Fe bond lengths, as expected from strongly delocalized valence changes (due to the soft S-ligands and π-backbonding to the CO and CN^−^ ligands). More specifically, the addition of cyanide ligands to the parent hexacarbonyl complex 6, to yield 2^2−^, resulted in an upshift of approximately 0.2 eV in Fe K-edge energy. This shift was fully reversed following the addition of the BCF to yield 3^2−^, in agreement with the hypsochromic shift observed in FTIR (see Fig. 2 and Table 1). A small K-edge upshift (∼0.1 eV) was also found for the conversion of 3^2−^ to the μ-hydride species 4^−^, although the formal iron oxidation state increased by two units in the diferrous species. However, a hypsochromic shift of 80–100 cm^−1^ in the carbonyl vibrations of 4^−^ was observed by FTIR. A similarly small K-edge shift and large IR band shift have previously been reported for the protonation of the di-phosphine analogue Fe_2_(μ-pdt)(CO)_4_(PMe_3_)_2._^45^ Also the increase of the Fe–Fe distance in the μ-hydride state by ∼0.04 Å from EXAFS is similar for both complexes.^57^ In part, the small K-edge shift may be explained by a shape change due to the conversion of 5-coordinated to 6-coordinated iron centers in the hydride complexes, possibly counteracting an oxidation-related shift. More importantly, for the phosphine complex, the formal μ-H^−^ ligand was shown by DFT to remain relatively protic in nature, with a Mulliken charge close to zero and charges at the iron ions that were even slightly more negative in the hydride state as well as significant surplus positive charge on the phosphines.^45^ The similar geometry change and a similar charge distribution here involving the CN-BCF ligands likely accounts for the XAS and FTIR properties of 4^−^.

**Table 1:** IR band frequencies of the studied diiron complexes (see Fig. 2)

Compound	Line colour	Wavenumbers/cm^−1^	Ref.
2^2−^	Black (Fig. 2)	*ṽ*_(CO)_ = 1965, 1924, 1886 *ṽ*_(CN)_ = 2071	This work and 46
3^2−^	Red (Fig. 2)	*ṽ*_(CO)_ = 1989, 1954, 1920 *ṽ*_(CN)_ = 2134	This work and 28
4^−^	Blue (Fig. 2)	*ṽ*_(CO)_ = 2070, 2050, 2020 *ṽ*_(CN)_ = 2186	This work and 28
5	Green (Fig. 2)	*ṽ*_(CO)_ = 2009, 1989, 1953 *ṽ*_(CN)_ = 2151	This work
6		*ṽ*_(CO)_ = 2074, 2033, 1994	38

In contrast, a more distinct up-shift of approximately 0.6 eV of the Fe K-edge energy was observed upon AgNO_3_ oxidation of 3^2−^ to form 5, in agreement with a more Fe centered oxidation. In particular the smaller Debye–Waller factor (*σ*) of the Fe–C(O) bonds from EXAFS suggests that partial degradation may lead to species with partial loss of the CO ligands of 3^2−^ in the oxidized sample, but the determined metal–ligand bond lengths and Fe–Fe distance (Table S4†) otherwise support a quite similar structure as for 3^2−^ in 5. Notably, reduction of 4^−^ with CoCp yielded a XANES and EXAFS spectrum that was very similar to the spectrum of 3^2−^ (Fig. 4B and E), supporting significant reversibility of the reaction, in agreement with the FTIR data.

### Cyclic voltammetry of 3^2−^ and 4^−^

**Fig. 5:**
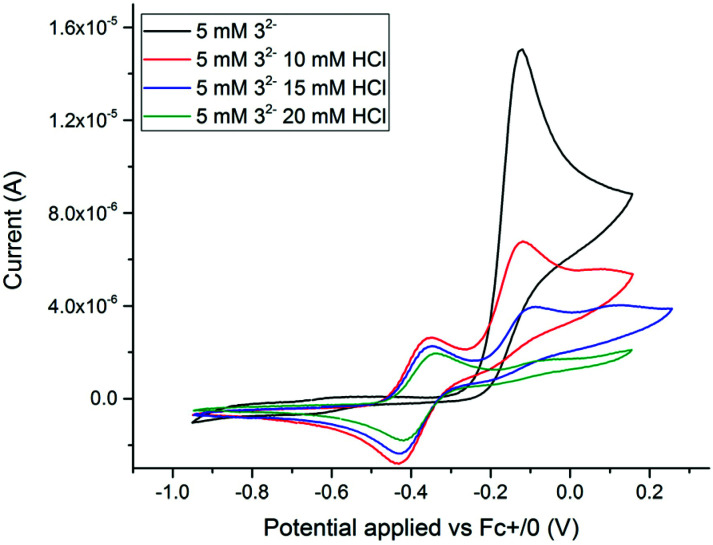
Cyclic voltammograms showing oxidation features of complexes 3^2−^ and 4^−^ in acetonitrile. Complex 4^−^ is generated *in situ via* addition of HCl to a solution of 3^2−^. 5 mM analyte, 0.2 M TBAPF_6_ (electrolyte), scan rate: 0.1 V s^−1^, scan window: −1.0 to 0.2 V *vs.* Fc^+/0^.

**Fig. 6:**
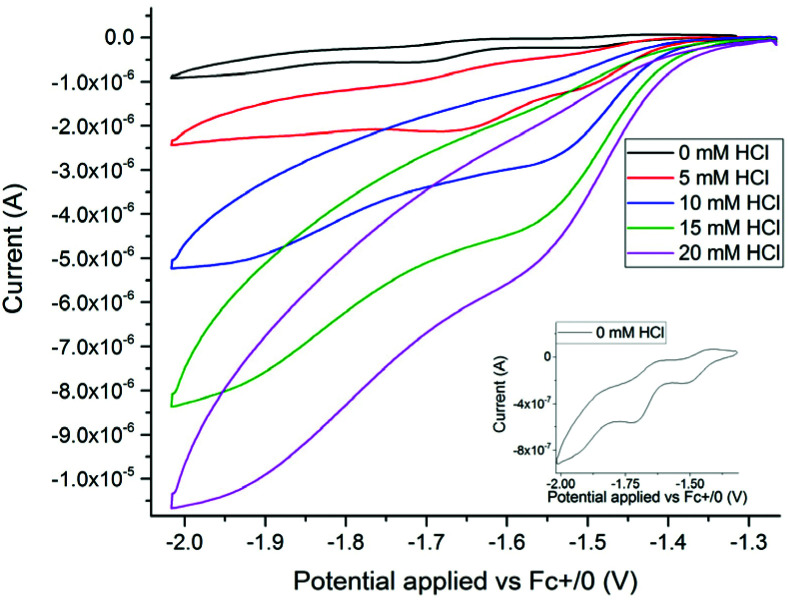
Cyclic voltammograms showing the catalytic current response observed when adding HCl to 3^2−^ (5 mM) in acetonitrile. The first five titration points are shown; 0 mM HCl (black trace, also in inset); 5 mM HCl (red trace); 10 mM HCl (blue trace); 15 mM HCl (green trace); 20 mM HCl (purple trace). The full titration is shown in the ESI Fig. S22.† 0.2 M TBAPF_6_ (electrolyte), scan rate: 0.1 V s^−1^, scan window: −1.25 to −2.01 V *vs.* Fc^+/0^.

Electrochemical properties of 3^2−^ and 4^−^ were studied by cyclic voltammetry (CV). All redox events are quoted against a ferrocene internal reference unless otherwise stated. *In situ* generation of 4^−^ was achieved *via* addition of HCl (in Et_2_O) to a solution of 3^2−^. As observed in Fig. 5 and 6, formation of 4^−^ resulted in significant differences in both the oxidation and reduction processes.

### Electrochemical oxidation of 3^2−^ and 4^−^

CVs of 3^2−^ in acetonitrile reveals an irreversible oxidation event at *E*_p_ = −0.12 V, which was attributed to an [Fe(i)Fe(i)]/[Fe(i)Fe(ii)] oxidation. This differs from the quasi-reversible redox couple at *E*_1/2_ −0.3 V observed in dichloromethane by Manor *et al.* and reproduced by us (ESI_8, Fig. S13†). The electrochemically irreversible nature of the oxidation of 3^2−^ is in agreement with the spectroscopically observed instability of 3^−^ and formation of 5.

Stepwise addition of HCl to 3^2−^ to give 4^−^, caused a change in the CV. Addition of 2 eq. HCl resulted in the appearance of a new quasi-reversible redox couple at −0.48 V (Fig. 5; ESI_5–8, Fig. S10–13†). Upon further addition of HCl, the quasi-reversible redox event shifted another 20 mV in the positive direction (−0.46 V) and became more defined (Fig. 5). This change in the cyclic voltamogram is in agreement with the slight stoichiometric excess of HCl required to cause the structural change from 3^2−^ to 4^−^ observed by FTIR and UV-vis spectroscopy (Fig. 2 and ESI_1, ESI_2†). Analysis of a ferrocene reference indicated that the peak separation of a reversible couple under our cell conditions is 81 mV (ESI_5, Fig. S10†). We thus assign the new redox event to the reversible oxidation of 4^−^ from [Fe(ii)Fe(ii)] to [Fe(ii)Fe(iii)]. To confirm the homogeneity of this process, CVs were recorded at increasing scan rates 50–5000 mV s^−1^. The peak anodic (*i*_p,a_) and peak cathodic (*i*_p,c_) currents of the reversible oxidation event were analysed in a Randles–Sevcik plot (ESI_6, Fig. S11†). The linear dependence of *i*_p,a_ and *i*_p,c_ on the square root of the scan rate demonstrates that oxidation of 4^−^ is indeed a diffusion-controlled process with a diffusion coefficient of ∼4 × 10^−5^ cm^2^ s^−1^. Trumpet plot analysis was carried out to determine a heterogeneous electron transfer rate constant of ∼0.011 cm s^−1^ (ESI_7, Fig. S12†). The obtained rate constant may also serve as an estimate for the rate constants of the other heterogeneous electron transfer steps in the catalytic cycle.^58–62^

### Electrocatalytic reduction of protons

In the absence of protons, two quasi-reversible reduction events are observable for 3^2−^, occurring at *E*_p,1_ = −1.51 V and *E*_p,2_ = −1.71 V (Fig. 6, inset). Titration of HCl causes the onset potential of reduction of 3^2−^ to shift to more positive values (*i.e.*, from *ca.* −1.4 V to −1.25 V) while also significantly increasing the current amplitude (Fig. 6). This shift in onset potential is in accordance with the bridging hydride, 4^−^, being a catalytically relevant species. The catalytic current continued to increase up to the addition of approximately 20 eqs of HCl (relative to 3^2−^, final HCl concentration 0.1 M, ESI_15, Fig. S22†), at which point the titration was stopped to retain the molecular integrity of 4^−^ (ESI_1, Fig. S2†). Control experiments shown in Fig. S23† demonstrate that the observed large catalytic current does indeed originate from complex 3^2−^/4^−^. Within the studied potential range, the catalytic current observed at strongly reducing conditions (*i*_p_ at −1.95 V) was observed to vary linearly with HCl concentration, whereas the current at ≈ −1.50 V displayed a more complex HCl dependence (ESI_16, Fig. S24 and S25†). At the milder potential the current begins to plateau at an HCl concentration of 70–80 mM (14–16 eq. relative to 3^2−^). However, the close proximity to the second catalytic wave prohibits full quantitative analysis of the limiting current (*i*_lim_).

Considering the amplitude of the reduction current and its strong dependence on acid concentration, we attribute these processes to electrocatalytic proton reduction. Based on the spectroscopically observed reactivity of 3^2−^ towards protons, a possible catalytic mechanism involves H_2_ formation proceeding *via* initial protonation. Subsequent reduction of 4^−^ yields the reduced hydride 4^2−^ as an intermediate, as observed upon treatment of 4^−^ with CoCp* by EPR spectroscopy. Thus, the first two steps of the catalytic cycle can be summarized as a CE type mechanism, where C denotes a chemical step (protonation) and E refers to a redox event (reduction). The order of the second redox and chemical steps is more elusive. The differences in current response to HCl concentration at −1.5 and −1.95 V suggests that two different catalytic pathways can be accessed as a function of potential, with a slower catalytic cycle operating at the milder potential. The latter can be rationalized as a CECE mechanism where 4^2−^ is a sluggish hydride donor towards HCl. Under more reducing conditions, we consider a second reduction to give 4^3−^ a more plausible pathway, and the reaction between 4^3−^ and a second proton gives 3^2−^ and H_2_ may close the catalytic cycle, *i.e.* a CEEC type mechanism. The latter step potentially involves formation of a transient di-hydride species, with H–H bond formation occurring *via* homolytic reaction.^63,64^ A summary of the observed and proposed reaction steps is provided in Scheme 1. We note that a parallel catalytic cycle proceeding *via*3^−^ can become available at more reducing potentials, however it is omitted from Scheme 1 for clarity.

## Conclusions

In summary, the present study explores the importance of protecting the cyanide ligands for the catalytic function of cyanide containing [2Fe]_H_ subsite mimics. The addition of Lewis acids stabilizes complex 2^2−^ under acidic conditions, enabling electrocatalytic proton reduction. Clearly this reflects an important factor in rationalizing why complex 1^2−^ and, to a limited extent, 2^2−^ become catalytically active upon insertion into the active-site of [FeFe] hydrogenase.^65,66^ Here, the cyanide ligand capping approach has allowed the observation of catalytically relevant iron oxidation states of the [2Fe]_H_ subsite, *i.e.* Fe(i)Fe(i) and Fe(ii)Fe(ii), *via* UV-Vis, FTIR, and XAS; while EPR studies suggest the formation of short-lived mixed valent Fe(i)Fe(ii) intermediates. Albeit the corresponding oxidation states have previously been observed in phosphine ligated analogous, this paves the way for their detailed characterization also in the biologically more relevant cyanide ligated diiron complexes. From the combined electrochemical and spectroscopy data we propose that the catalytic cycle(s) of 3^2−^ operates at the same oxidation state levels as the [2Fe]_H_ subsite, but proceeds *via* bridging hydride intermediates. Detailed kinetic analysis of the (electro)catalytic cycle is currently underway. Moreover, in light of the current debate concerning the formation of bridging hydrides in the H-cluster, it is noteworthy that the μ-hydride species 4^−^ can form *via* direct protonation of the Fe–Fe bond in 3^2−^, *i.e.* without involving a terminal hydride intermediate.

Exploring the possibility to fine-tune the protonation and redox properties of these di-cyanide complexes *via* variations of the Lewis acid is a promising theme for future studies. In order to further improve the relevance of these complexes as mechanistic and spectroscopic models, parallel efforts need to be directed at stabilizing the rotated structure in order to promote terminal hydride formation, potentially achievable *via* introduction of steric bulk on the bridging di-thiolate ligand or asymmetric ligand substitution. In addition to providing suitable spectroscopic models,^67,68^ the cyanide ligands also provide possible binding sites in the preparation of electrocatalytic polymers, such as metal–organic frameworks.^69^

## Author contributions

Holly J. Redman: formal analysis, investigation, methodology, validation, visualisation, conceptualisation, writing (original draft). Ping Huang: EPR simulation and discussions. Michael Haumann: XAS experiments, writing (review and editing). Mun Hon Cheah: resources, supervision, validation, writing (review and editing). Gustav Berggren: resources, supervision, validation, project administration, funding acquisition, writing (review and editing).

## Conflicts of interest

There are no conflicts to declare.

## Supplementary Material

DT-051-D1DT03896F-s001
