# Supplementary material for: Lewis acid protection turns cyanide containing [FeFe]-hydrogenase mimics into proton reduction catalysts
Source: Dalton Trans. 2022 Feb 18;51(12):4634–43. doi: 10.1039/d1dt03896f (PMC8939051; doi:10.1039/d1dt03896f)
Supplement: DT-051-D1DT03896F-s001 [file DT-051-D1DT03896F-s001.pdf]

Electronic Supplementary Material (ESI) for Dalton Transactions.
This journal is © The Royal Society of Chemistry 2022

Lewis acid protection turns cyanide containing [FeFe]-hydrogenase mimics into proton reduction catalysts

Holly J. Redman[1], Ping Huang[1], Michael Haumann[2], Mun Hon Cheah[1], Gustav Berggren[1,*]

[1] Department of Chemistry – Ångström Laboratory, Uppsala University, Box 523, 75120 Uppsala, Sweden
E-mail: gustav.berggren@kemi.uu.se

[2] Department of Physics, Freie Universität Berlin, Arnimallee 14, 14195 Berlin, Germany

Contents:

SI_1 Titration of HCl into complex 3^{2-} monitored by FTIR spectroscopy 2
SI_2 Titration of HCl into complex 3^{2-} monitored by UV-Vis spectroscopy 3
SI_3 Treatment of complex 3^{2-} with DCl 5
SI_4 Determination of protonation rate constant by stopped flow rapid mixing FTIR 6
Experimental procedure 6
Rapid scan OPUS program settings 6
Example of raw spectra collected 7
Baseline treatment 8
Kinetic analysis 8
Fit data 9
Second order rate constant 10
SI_5 Determination of peak separation of $Fc^{+/0}$ as an internal reference 11
SI_6 Randles Sevcik analysis of oxidation of complex 4^{-} 12
SI_7 Trumpet plot analysis of oxidation of complex 4^{-} 14
SI_8 Cyclic voltamogram of 3^{2-} in dichloromethane 15
SI_9 Titration of $AgNO_3$ into complex 3^{2-} monitored by UV-vis spectroscopy 16
SI_10 Titration of $AgNO_3$ into complex 3^{2-} monitored by FTIR spectroscopy 16
SI_11 Reduction of 5 by $NaBH_4$ or CoCp* observed by FTIR spectroscopy 17
SI_12 EPR spectra recorded of 3^{2-} oxidized by $AgNO_3$ at room temperature and –70 °C to make 5 and 3^{-} respectively 17
SI 13 EPR spectra of 4^{-} and CoCp* mixed at -40 °C, power and temperature dependence, and simulations . 18
SI_ 15 cyclic voltammetry of 3^{2-}, full titration with HCl 21
SI_16 Peak current plotted against concentration of HCl at -1.95 V and -1.55 V vs Fc+/0 22
SI_17 X-ray absorption spectroscopy 23
SI_references 25

SI_1 Titration of HCl into complex 3^{2-} monitored by FTIR spectroscopy

A solution of **3**$^{2-}$ was prepared (0.5 mM, 5 mL) and aliquoted into 500 µL portions. Another solution of HCl (1 M in Et_2O diluted to 25 mM in MeCN) was prepared. This solution was titrated into each aliquot of **3**$^{2-}$ as follows; 5 µL (0.5 eq); 10 µL (1 eq); 15 µL (1.5 eq); 20 µL (2 eq); 25 µL (2.5 eq); 30 µL (3 eq); 35 µL (3.5 eq); 40 µL (4 eq). Each titration point was monitored by UV-vis spectroscopy, the end point was recorded by UV-vis and FTIR spectroscopy. End point IR (MeCN) ṽ/cm^{-1} = 2020, 2050, 2070, 2186.

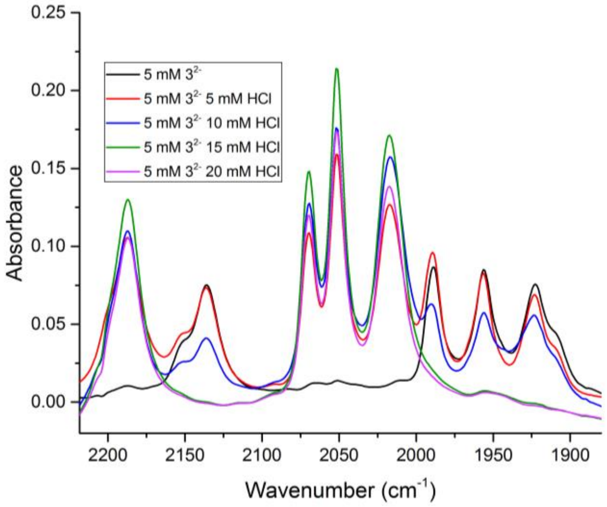

Figure S1. FTIR spectrum of **3**$^{2-}$ (5 mM, black spectrum) and formation of complex **4**$^{-}$ (purple spectrum) through titration of HCl. **4**$^{-}$ is visible in the FTIR spectrum after addition of 1 or 2 equivalents of HCl (red and blue spectra respectively), demonstrating that no or a small excess of HCl already forms **4**$^{-}$ near-quantitatively. Full conversion is achieved after addition of 3 equivalents of HCl (green spectrum).

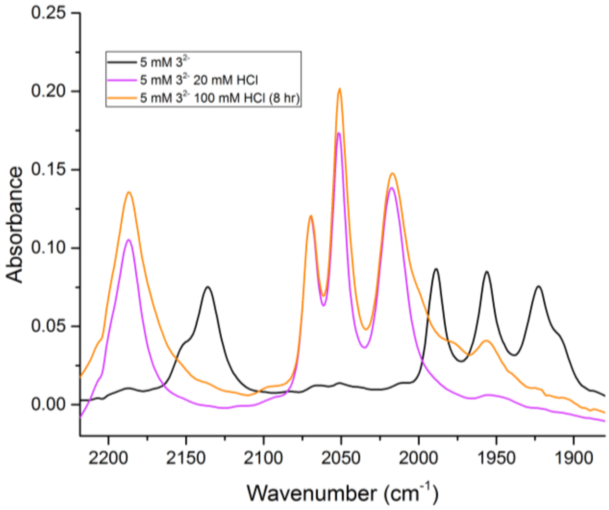

Figure S2. FTIR spectrum of **3**$^{2-}$ (5 mM, black spectrum) and formation of complex **4**$^{-}$ (purple spectrum) through titration of HCl. The spectra demonstrate that complex **4**$^{-}$ is stable even at high HCl concentrations and after 8 hours of cyclic voltammetry experiments at 20 eq HCl (100 mM, orange spectrum).

SI_2 Titration of HCl into complex 3^{2-} monitored by UV-Vis spectroscopy

A solution of **3^{2-}** was prepared (0.5 mM, 5 mL) and aliquoted into 500 µL portions. Another solution of HCl (1 M in Et_2O diluted to 25 mM in MeCN) was prepared. This solution was titrated into each aliquot of **3^{2-}** as follows; 5 µL (0.5 eq); 10 µL (1 eq); 15 µL (1.5 eq); 20 µL (2 eq); 25 µL (2.5 eq); 30 µL (3 eq); 35 µL (3.5 eq); 40 µL (4 eq). Each titration point was monitored by UV-vis, the end point was recorded by UV-vis and FTIR spectroscopy. Start point UV-Vis λ_{max}/nm = 346 (abs = 1.98, $\varepsilon 3^{2-}$ = 3960 L mol^{-1} cm^{-1}); End point UV-Vis λ_{max}/nm = 331 (abs = 0.5, $\varepsilon 4^{-}$ = 1000 L mol^{-1} cm^{-1}).

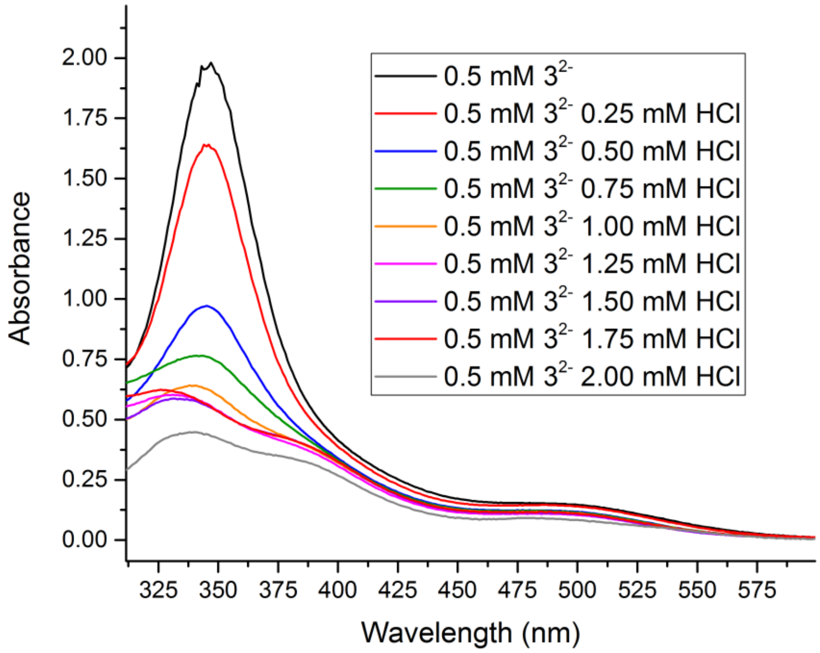

Figure S3. UV-vis spectra of titration points when HCl was added to a 0.5 mM solution of complex **3^{2-}** (black line) to give complex **4^{-}** (dark grey spectrum).

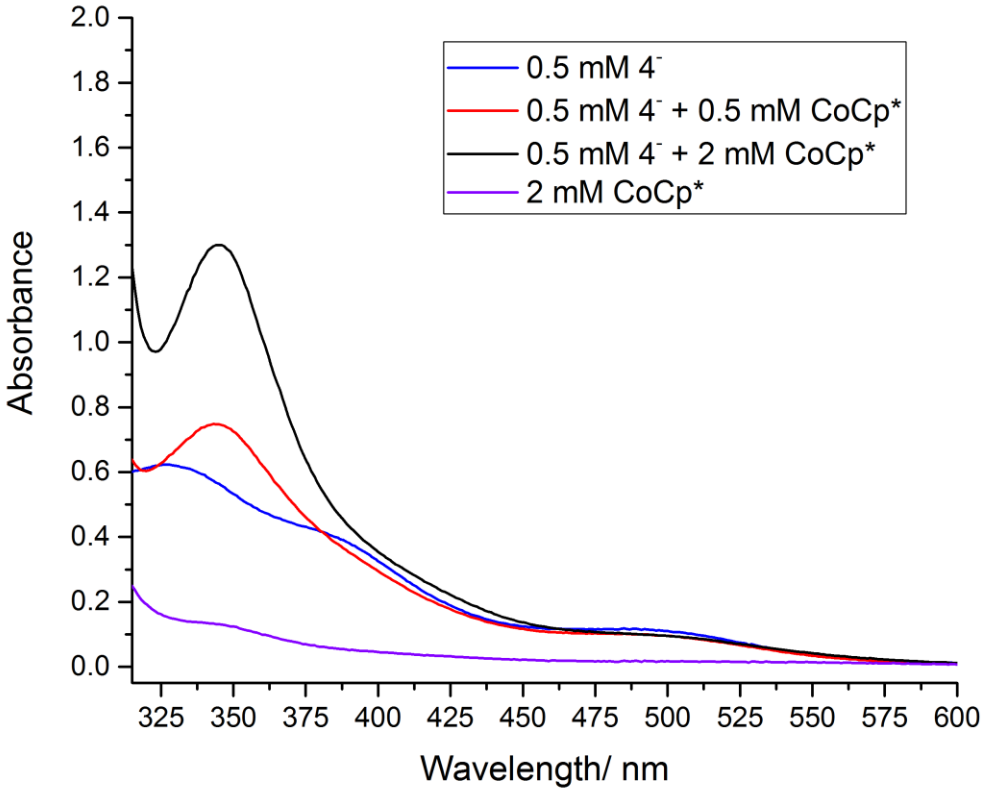

Figure S4. UV-Vis spectra showing **3**$^{2-}$ (black spectrum); the response of **4**$^{-}$ upon addition of 4 equivalents of CoCp*(red spectrum) (E = -1.94 vs Fc+/0)[1]. The absorbance at 346 nm is partially recovered following reduction of **4**$^{-}$; **4**$^{-}$ (blue spectrum); CoCp* (purple spectrum).

Table S1. Change in maximum absorbance at 346 nm upon addition of CoCp* to complex **4**$^{-}$.

**Sample**	**Absorbance at 346 nm**
**3**$^{2-}$ 0.5 mM	1.98
**3**$^{2-}$ 0.5 mM + 4 eq HCl	0.34
**3**$^{2-}$ 0.5 mM + 4 eq HCl + 4 eq CoCp*	1.30
CoCp* 2 mM	0.10

$$\frac{Abs_{(32-)CoCp*}}{Abs_{(32-)initial}} \times 100 = \frac{1.30}{1.98} \times 100 = 66\%$$

SI_3 Treatment of complex 3^{2-} with DCl

To a 3 mM solution of complex **3**$^{2-}$ in acetonitrile, 2 equivalents of DCl were added. There was no difference in the FTIR of complex **4**$^{-}$ and the isotopologue (i.e., 3^{2-}[μ-D]).

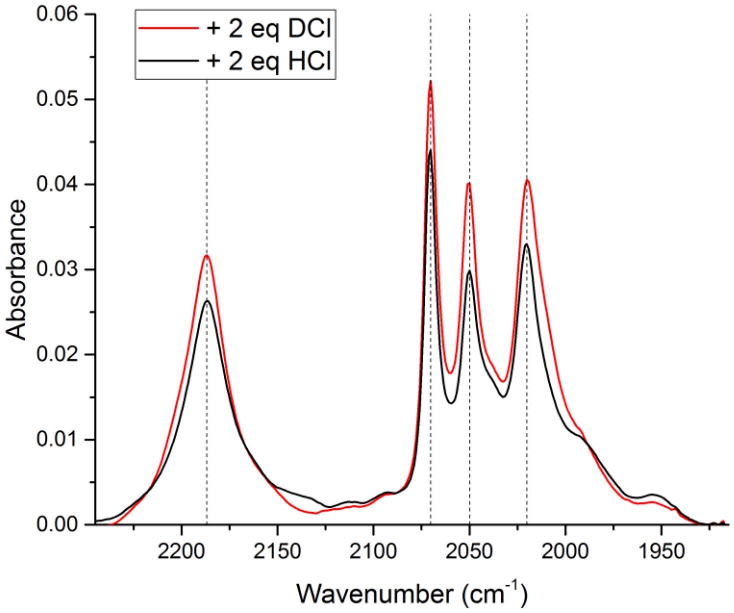

Figure S5. FTIR spectra of 3 mM **4**$^{-}$ (black spectrum) and **3**$^{2-}$ [μ-D] (red spectrum) in acetonitrile, showing no difference in the positions of the carbonyl or cyanide bands between the hydride and the deuteride species.

Table S2. Positions of carbonyl and cyanide bands (in cm^{-1}) derived from Fig. S5.

**3**$^{2-}$ + 2 eq HCl	**3**$^{2-}$ + 2eq DCl
2186	2186
2070	2070
2049	2049
2020	2020

SI_4 Determination of protonation rate constant by stopped flow rapid mixing FTIR

Experimental procedure

Stopped-flow rapid-mixing rapid-scan FTIR was used to monitor the protonation reaction of $\mathbf{3}^{2-}$ to form $\mathbf{4}^{-}$. In the glovebox, one syringe was filled with a 0.1 mM solution of $\mathbf{3}^{2-}$ in acetonitrile, making sure to remove all gas bubbles from the syringe. The second syringe was loaded with a solution of HCl. The HCl solution had been diluted from 1 M HCl in diethyl ether to yield x mM solutions. Where x = 0.4, 0.6, 0.8 or 1.6. The FTIR cell was filled with acetonitrile. The stopped flow apparatus was removed from the glovebox and brought to the FTIR spectrometer. The FTIR cell was put in the sample chamber which was subsequently purged with nitrogen for 10 minutes before collecting a background spectrum.

Next, the syringes were manually compressed using the push-plate. The contents of both syringes travelled to the FTIR cell and were mixed. The contents of the FTIR cell from the previous measurement were transferred to the waste syringe pushing the trigger so that the control software could start the rapid scan measurement. Due to the large size of the sample syringes compared to the waste syringe, it was possible to measure up to 5 repeats from the same syringes.

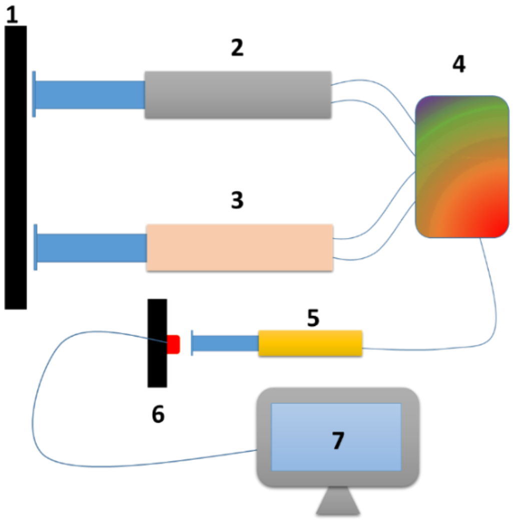

1. Push plate
2. 2.5 cm³ syringe containing 0.1 mM complex 3 in MeCN
3. 2.5 cm³ syringe conaining x mM HCl where x = 0.4, 0.6, 0.8, 1.6. diluted from 1 M HCl in Et2O using MeCN
4. FTIR Cell inside spectrometer sample chamber
5. 1000 µL waste collection syringe
6. Trigger plate – informs the program to start the rapid scan measurement
7. Computer – contains OPUS program for rapid scan FTIR

Figure S6. Schematic diagram of the stopped-flow experimental set-up used for the rapid mixing protonation experiments. Syringe 2 holds 0.1 mM $\mathbf{3}^{2-}$ in MeCN and syringe 3^{2-} holds the x mM HCl where x = 0.4, 0.6, 0.8, 1.6. When the syringes are compressed using the push plate (1), the reactants travel through the tubing to the FTIR cell where they are mixed. The excess solution goes into the waste syringe (5) and this pushes the trigger plate (6) which then triggers the instrument software to measure IR spectra for monitoring of the reaction. The deadtime of the instrument is ~50 ms and the time resolution is also ~50 ms.

Rapid scan OPUS program settings

Basic:

Experiment HJR_RS_MCT.xpm; Sample description 0p1mMpdtBCF_XmMHCl; sample form liquid; Path n/a

Method editor:

Description set buffer 2; wait input high 7; start loop 150; measure 1; end loop; use buffer 1 as background; split double sided interferogram.

Advanced:

Resolution 2 cm^{-1}; sample scan time 4 scans; background scan time 4 scans; save data 2750 – 1250 cm^{-1}; result spectrum absorbance; interferogram size 4738 points; FT size 8K; data blocks to be saved absorbance, single channel, background.

Optic:

Source setting MIR; beam splitter KBr; optical filter setting open; aperture setting 2.0 mm; accessory any; measurement channel sample compartment; background measurement channel sample compartment; detector setting LN-MCT photovoltaic [internal pos.2]; scanner velocity 160 kHz; sample signal gain automatic; background signal gain automatic; delay after device change 0 sec; delay after measurement 0 sec; optical bench ready OFF.

Acquisition:

Wanted high frequency limit 4000 cm^{-1} (5266.04 cm^{-1}); wanted low frequency limit 0 cm^{-1} (0.0 cm^{-1}); laser wavenumber 15798.12; high pass filter open; low pass filter open; acquisition mode double-sided, forward-backward; correlation mode OFF; external analog signals OFF.

FT:

Phase resolution 16; phase correction mode Mertz; Apodization function Blackman-harris 3-term; zerofilling factor 2; interferogram size 4738 points; FT size 8 K; perform interferogram non-linearity correction before FT.

Example of raw spectra collected

The carbonyl ligands of **3**$^{2-}$ were easily visible at 1922 cm^{-1}, 1956 cm^{-1}, and 1988 cm^{-1} (Fig. S7, thick blue spectrum). At the end of the rapid scan experiment, the carbonyl bands of **4**$^{-}$ are also visible (Fig. S7, thick magenta spectrum). The region containing the bands resulting from **4**$^{-}$ was partially obscurred in earlier time measurements by a large broad artefact at 2070 cm^{-1} that we attribute to a change in the path length caused by mixing of the solutions. The band at 2070 cm^{-1} is present in our acetonitrile background spectra and is observed in all experiments including a rapid mixing experiment where both syringes contained only acetonitrile. This complicated a detailed kinetic analysis of the appearance of **4**$^{-}$ and the rates were instead primarily based on the disappearance of the spectral features of **3**$^{2-}$.

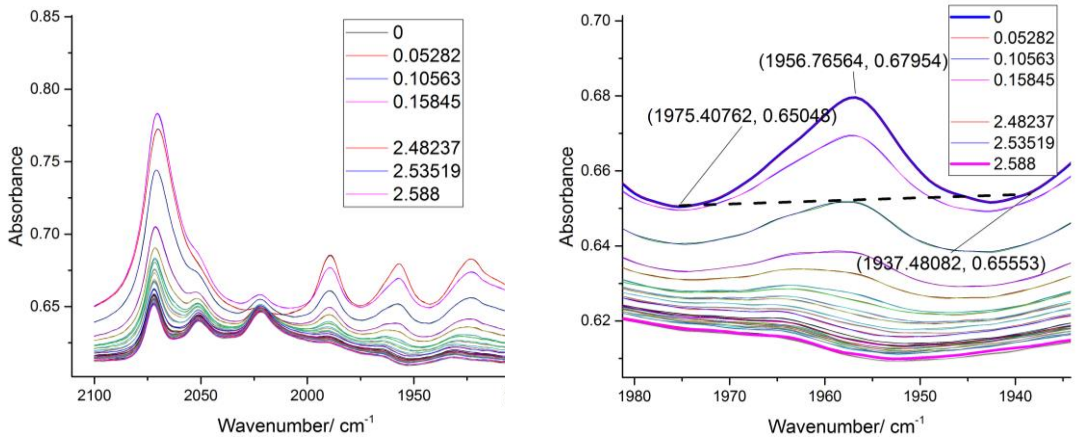

Figure S7. Left: Examples of raw spectra collected from the stopped-flow rapid scan experiment. 0.1 mM complex **3**$^{2-}$ and 0.8 mM HCl in MeCN. Right: Zoom into the graph on the left showing how the baseline was determined between 1975 cm^{-1} and 1937 cm^{-1}. Start and end spectra are highlighted (thick blue and magenta spectra, respectively)

Baseline treatment

A baseline shift is observed during the experiment. Thus, prior to kinetic analysis we performed a baseline treatment for each individual carbonyl band. In order to carry out the baseline treatment, a carbonyl band was selected to be analysed, for example 1956 cm^{-1}. The two points equidistant on either side of the peak at 1956 cm^{-1} were also selected. A representative example using the peak at 1956 cm^{-1} is shown in Fig. S8, using 1975 cm^{-1} and 1937 cm^{-1} for baseline estimates.

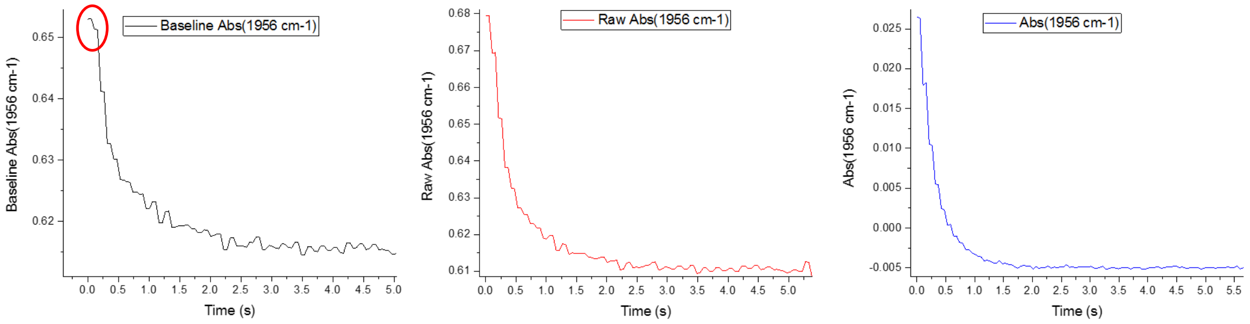

Figure S8. Left: graph of baseline absorbance at 1956 cm^{-1} against time to illustrate that the baseline does shift over time. Middle: Raw absorbance at 1956 cm^{-1} over time. Right: Baseline corrected absorbance at 1956 cm^{-1} over time produced by subtracting left graph from middle graph. Red circle highlight that there is very little change between the first and second point. Therefore the first point was omitted from all fitting data.

Kinetic analysis

Attempts to fit the resulting data for a pseudo first order chemical reaction were unsuccessful. Consequently, we carried out a second order analysis to determine a second order rate constant.

$$[A]_0 = x[B]_0$$

Where x = 4, 6, 8 or 16

$$[Abs] = [Abs]_0 - \frac{[HCl]_0[Abs]_0(1 - e^{([Abs]_0 - [HCl]_0)k_1 t})}{[HCl]_0 - [Abs]_0(e^{([Abs]_0 - [HCl]_0)k_1 t})}$$

Where:

$$[HCl]_0 = x[Abs]_0$$

This equation was inserted into the fitting function in the Origin program. SI figure 8 (right) shows that the difference between the first and second points is negligible. Therefore the first point was removed in order to improve the reliability of the fit. Only the initial 8 points of the reaction were fitted in order to plot the initial rate.

This analysis was carried out for each carbonyl band of **3**$^{2-}$ (i.e. 1922, 1956 and 1988 cm^{-1}) and for each concentration of HCl (i.e 4mM, 6 mM, 8 mM, 16 mM). Each measurement was repeated at least twice.

Fit data

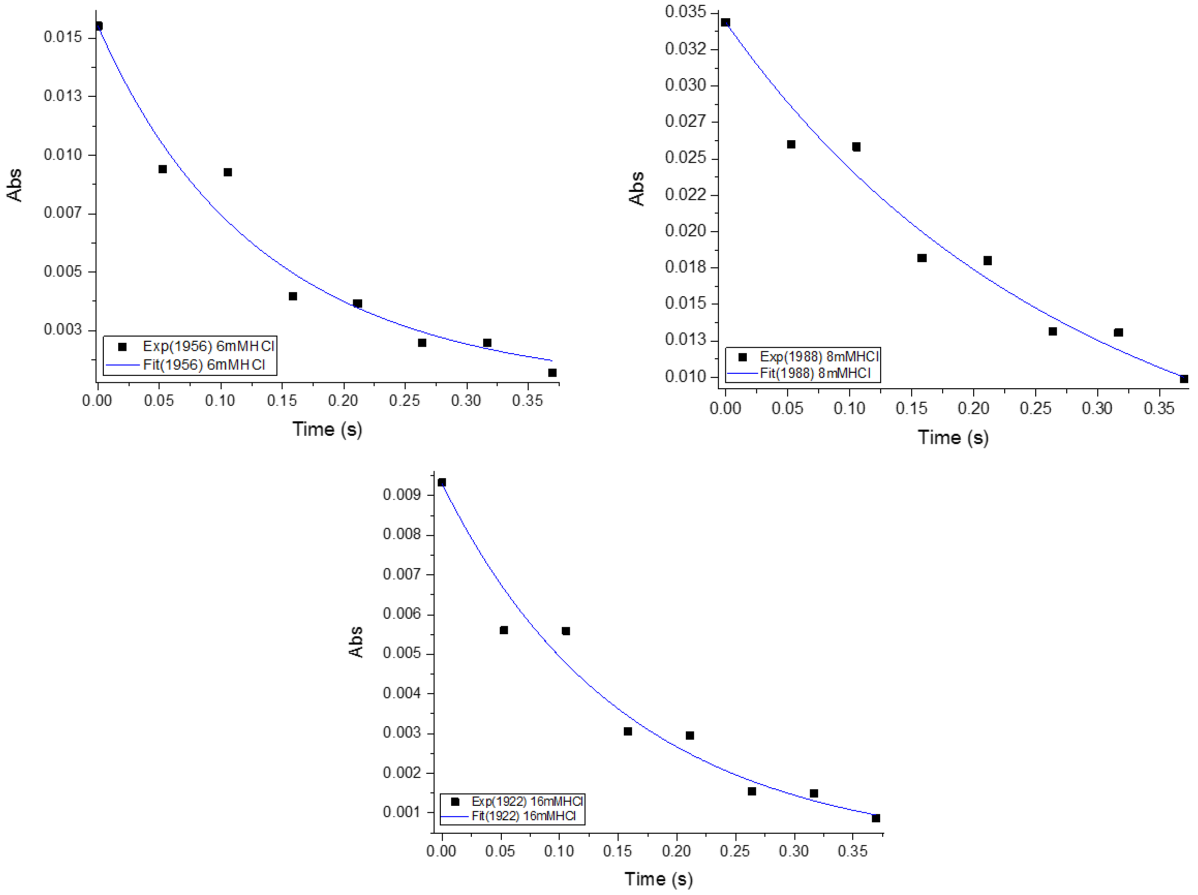

Figure S9. Three representative time traces taken from the stopped flow data and used to extract second order rate constants. The rate constants for all of the data collected are given below in Table S5.

Second order rate constant

Table S3. Compilation of second order rate constants and mean value.

Experiment	Wavenumber / cm^{-1}	Second order rate constant (k_1) / $L\ mol^{-1}\ s^{-1}$
4_mM _HCl_1	1922	11.5±0.7
4_mM_HCl_2	1922	13.1±0.9
6_mM_HCl_1	1922	13.2±0.8
8_mM_HCl_1	1922	23.0±2.1
8_mM_HCl_2	1922	16.1±0.9
16_mM_HCl_1	1922	28.5±3.1
4_mM _HCl_1	1956	10.8±0.7
4_mM_HCl_2	1956	12.6±0.8
6_mM_HCl_1	1956	15.7±1.2
8_mM_HCl_2	1956	25.0±1.9
16_mM_HCl_2	1956	15.1±1.1
4_mM _HCl_1	1988	9.9±0.5
4_mM_HCl_2	1988	11.6±0.7
6_mM_HCl_1	1988	11.3±0.7
8_mM_HCl_1	1988	19.3±1.7
8_mM_HCl_2	1988	12.8±0.7
16_mM_HCl_1	1988	24.5±2.7
Mean average value of k_1 / $L\ mol^{-1}\ s^{-1}$ (±SD)		16±6

SI_5 Determination of peak separation of $Fc^{+/0}$ as an internal reference

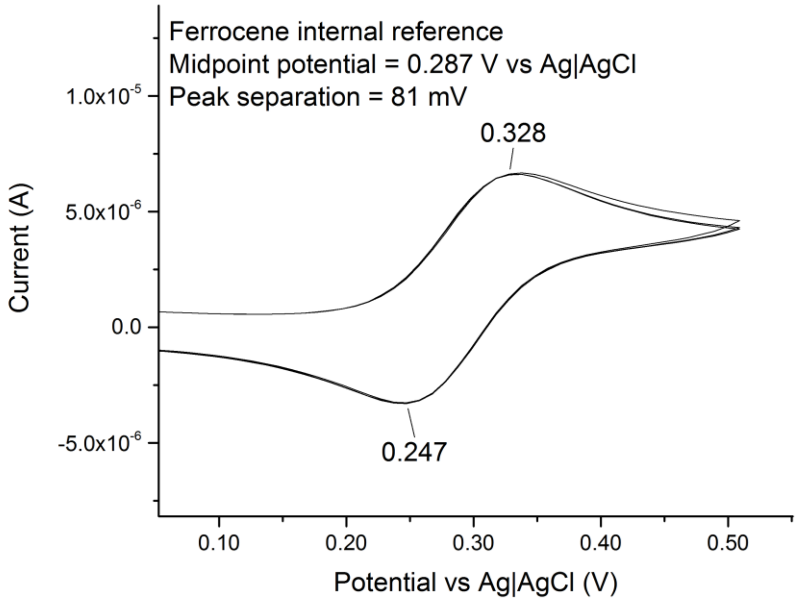

Figure S10. Cyclic voltammogram of ferrocene, showing how the internal reference potential was determined and also demonstrating that the well-known reversible couple has a peak separation of 81 mV in acetonitrile in the employed system. The mean value of the two peak voltages corresponds to a midpoint potential of 0.29 V vs. Ag/AgCl.

SI_6 Randles Sevcik analysis of oxidation of complex 4⁻

For the Randles-Sevcik analysis of the oxidation of **4⁻** at −0.48 V vs $Fc^{+/0}$, blank CVs at various scan rates were collected as background data and CVs of the complex were also collected in a scan window of −0.2 — 0.8 V vs. $Fc^{+/0}$). The background CVs were subtracted from the CVs of complex **4⁻**.

For each CV, the peak and trough currents were recorded ($i_{p,ox}$ and $i_{p,red}$) and plotted against the square root of the scan rate. This gave a plot that was linearly dependent on the square root of the scan rate. The slope of the linear fit and the Randles-Sevcik equation (for 25 °C) allowed the determination of the diffusion coefficient of the complex.[2]

$$i_p = (2.69 * 10^5) n^{\frac{3}{2}} A D^{\frac{1}{2}} C v^{\frac{1}{2}}$$

In a graph of i_p vs (scan rate)^{½} the slope represents the ratio of the two variables:

$$m = \frac{i_p}{v^{\frac{1}{2}}} = (2.69 * 10^5) n^{\frac{3}{2}} A D^{\frac{1}{2}} C$$

$$D^{\frac{1}{2}} = \frac{m}{(2.69 * 10^5) n^{\frac{3}{2}} A C}$$

$$D = \sqrt{\frac{m}{(2.69 * 10^5) n^{\frac{3}{2}} A C}}$$

$$D = \sqrt{\frac{m}{10.55825}}$$

Where:

ip – peak current in amps (A) ($i_{p,ox}$ or $i_{p,red}$).

n- number of electrons transferred, 1

A – electrode area (assuming a smooth circular electrode surface, 0.00785 cm^2)

C – concentration of analyte, $5x10^{-3}$ mol dm^{-3}

D – diffusion coefficient cm^2 s^{-1}

v – scan rate V s^{-1}

Data from two independent experiments yielded a diffusion coefficient of 3.31 ± 0.03 x 10^{-5} cm^2 s^{-1}.

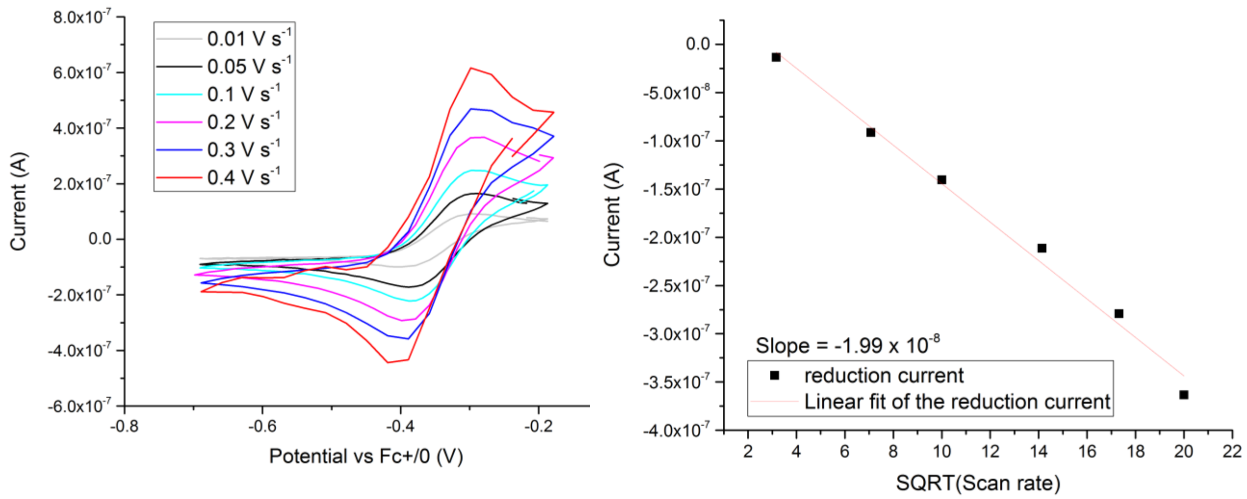

Figure S11. Representative example of the Randles-Sevcik analysis. Left: CVs of the reversible couple observed in **4**⁻ at -0.4 V vs. $Fc^{+/0}$ and recorded at scan rates ranging from 0.01 V s^{-1} to 0.4 V s^{-1}. Right: a plot of peak current against square root of scan rate. The slope of the linear fit (red line) was used to calculate the diffusion coefficient of **4**⁻.

SI_7 Trumpet plot analysis of oxidation of complex **4**$^{-}$

A Trumpet plot was used to calculate the standard rate constant for heterogeneous electron transfer (k_s). The peak and trough potentials of the oxidation wave of **4**$^{-}$ were recorded at various scan rates ($E_{p,ox}$ and $E_{p,red}$). Values for $E_{p,ox/red} - E_{½}$ were plotted against the log of the scan rate (log(v)). This plot was overlaid with a working curve generated in the electrochemistry fitting software (Digielch) using D_{sim} = 1 x10^{-5} cm^{2} s^{-1} and $k_{s,sim}$ = 1 cm s^{-1}. The x-axes of the two plots were shifted until the y-axes overlap. At this point the following holds true $\Lambda_{s,Fe} = \Lambda_{s,sim}$, where Λ_s is a dimensionless parameter defined in the following equation as:[2]

$$\Lambda_s = k_s \sqrt{\frac{RT}{FvD}}$$

Where:

k_s – heterogeneous rate constant

R – gas constant, 8.314 J K^{-s} mol^{-1}

F –Faraday constant, 96485.332 C mol^{-1}

D – Diffusion coefficient

At the overlap:

$$\Lambda_{s,sim} = \Lambda_{s,Fe} = k_{s,sim} \sqrt{\frac{RT}{FvD_{sim}}} = k_{s,Fe} \sqrt{\frac{RT}{FvD_{Fe}}}$$

F, R and T are all constant across the expressions:

$$k_{s,sim}\sqrt{D_{sim}} = k_{s,Fe}\sqrt{D_{Fe}}$$

It follows that:

$$\log\left(\frac{k_{s,sim}\sqrt{(D_{Fe})}}{k_{s,Fe}\sqrt{D_{sim}}}\right) = \frac{1}{2}(\log(v_{sim}) - \log(v_{Fe}))$$

$$\log\left(\frac{k_{s,sim}\sqrt{(D_{Fe})}}{k_{s,Fe}\sqrt{D_{sim}}}\right) = 1.650515$$

$$\log(k_{s,Fe}) = \log\left(\frac{k_{s,sim}\sqrt{D_{Fe}}}{\sqrt{D_{sim}}}\right) - 1.650515$$

$$\log(k_{s,Fe}) = -1.969$$

$$k_{s,Fe} = 10^{-1.969} = 0.011$$

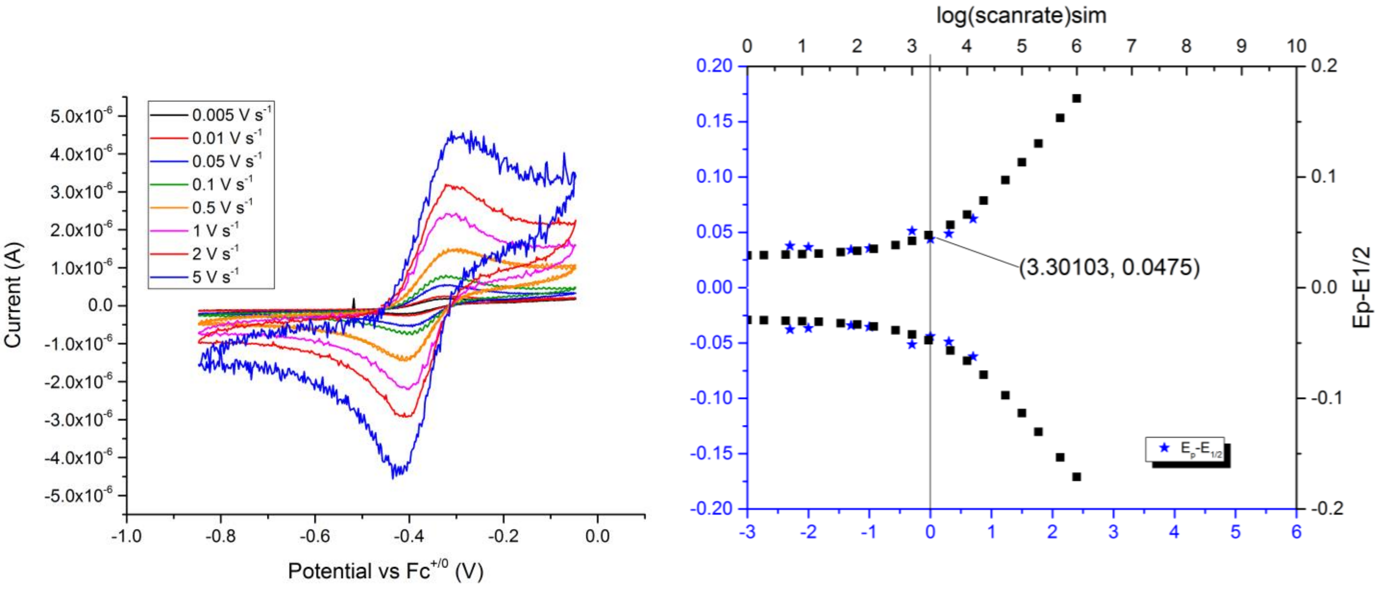

Figure S12. Cyclic voltammograms and Trumpet plot. Left: CVs of the reversible couple observed in **4**⁻ at -0.4 V vs. $Fc^{+/0}$ and recorded at scan rates ranging from 0.005 V s^{-1} to 5 V s^{-1}. Right: (magenta points) Trumpet plot of the difference between $E_{1/2}$ and the peak potentials E_a and E_c against the log of scan rate used to determine the heterogeneous electron transfer rate constant for this particular system. (grey points) Trumpet plot determined by simulating the cyclic voltammograms of an ideal reversible electrochemical couple. The two trumpet plots were overlaid and the x-axes compared to find ($\log(\nu_{sim})$-$\log(\nu_{fe})$).

SI_8 Cyclic voltamogram of 3^{2-} in dichloromethane

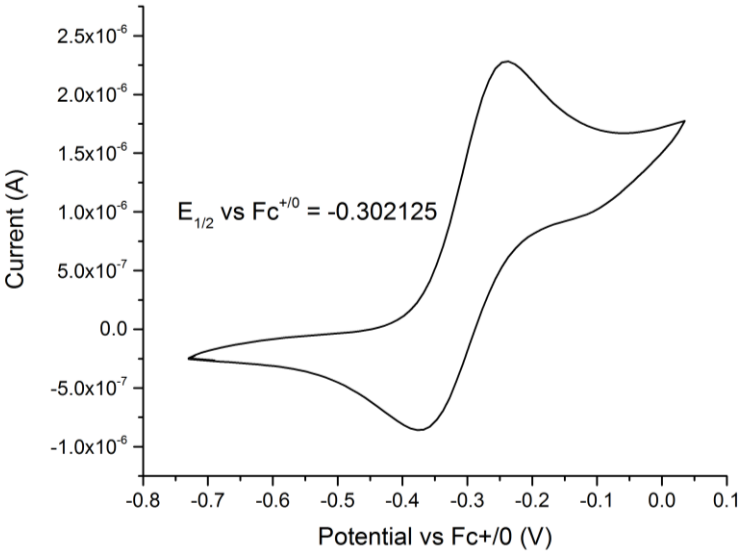

Figure S13. Cyclic voltammogram of complex $\mathbf{3}^{2-}$ (5 mM) measured in TBAPF6 (0.1 M) dissolved in dichloromethane (scan rate of 0.1 V s^{-1}). The CV demonstrates a pseudo-reversible redox couple at -0.302 V vs. Fc+/0, this is similar to the value observed by Manor et al. in 2014.[3] This pseudo reversible couple is not observed in our CV measurements of $\mathbf{3}^{2-}$ in acetonitrile (main text, Fig. 6).

SI_9 Titration of $AgNO_3$ into complex 3^{2-} monitored by UV-vis spectroscopy

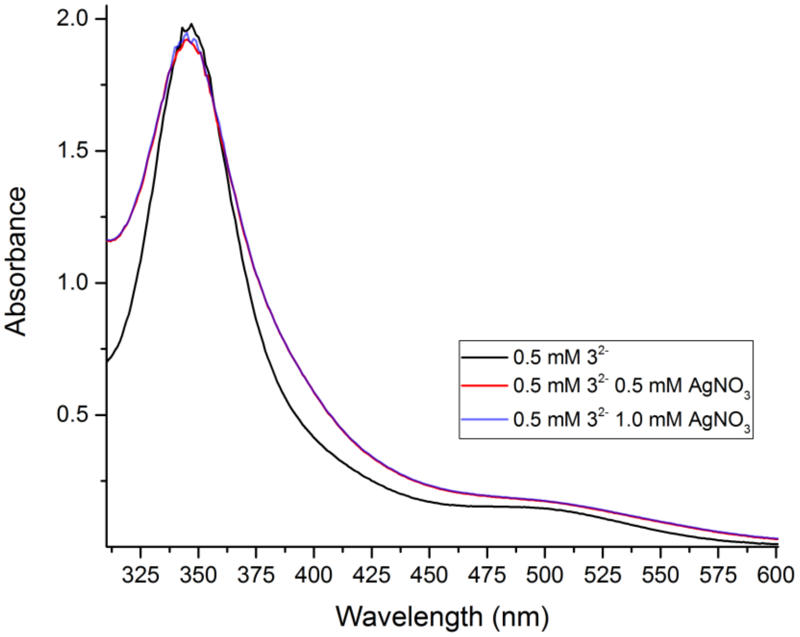

Figure S14. UV-vis spectra showing that when complex $\mathbf{3}^{2-}$ (0.5 mM, black spectrum) is oxidized to **5** (red and blue spectra) in MeCN by $AgNO_3$ the UV-vis spectrum broadens.

SI_10 Titration of $AgNO_3$ into complex 3^{2-} monitored by FTIR spectroscopy

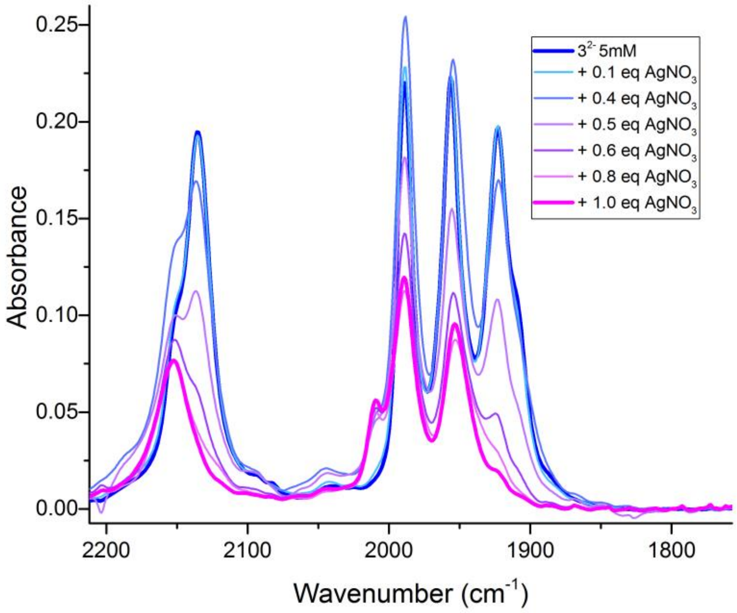

Figure S15. FTIR spectra demonstrate that only 1 eq of $AgNO_3$ is required to oxidise $\mathbf{3}^{2-}$ (5 mM, thick blue spectrum) to **5** (thick magenta spectrum). As Ag^+ is added to $\mathbf{3}^{2-}$ (thick blue spectrum), the FTIR signal begins to decrease and shifts by 40 cm^{-1} to higher wavenumbers, approximately 46 % of the signal amplitude at 1956 cm^{-1} is lost.

SI_11 Reduction of 5 by $NaBH_4$ or CoCp* observed by FTIR spectroscopy

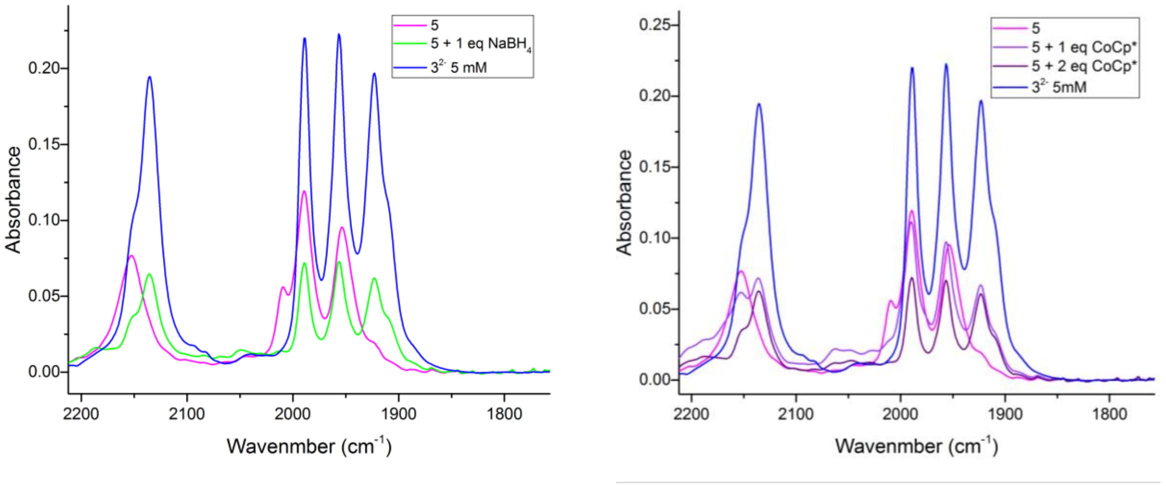

Figure S16. FTIR spectra in MeCN show that treatment of **5** (magenta spectrum) with $NaBH_4$ (left; green spectrum) or CoCp* (right; purple spectrum) results in partial recovery of complex $\mathbf{3}^{2-}$. After treatment with $NaBH_4$ and CoCp*, only 32 % of the original carbonyl signal of $\mathbf{3}^{2-}$ is recovered.

SI_12 EPR spectra recorded of 3^{2-} oxidized by $AgNO_3$ at room temperature and –70 °C to make 5 and 3^{-} respectively

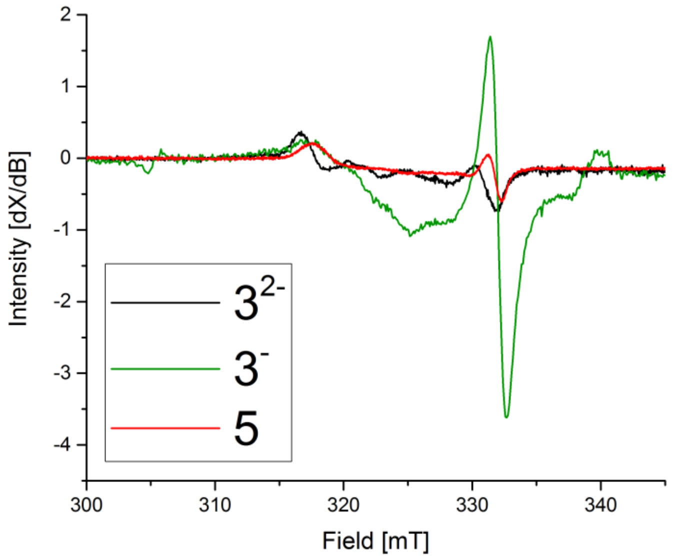

Figure S17. EPR spectra of 0.25 mM $\mathbf{3}^{2-}$ (black spectrum), mixed with $AgNO_3$ at room temperature to make **5** (red spectrum), and mixed with $AgNO_3$ at -70 °C to yield a species with an isotropic signal with g = 2.022 (green spectrum), tentatively attributed to $\mathbf{3}^{-}$. Spectra were measured at 10 K and 1 mW microwave power. The signal at ~317 mT is attributed to a small amount of an unknown EPR active substance in the starting material ($\mathbf{3}^{2-}$).

SI 13 EPR spectra of 4⁻ and CoCp* mixed at -40 ºC, power and temperature dependence, and simulations

Mixing of **4**⁻ with CoCp* at -40 ºC resulted in a complicated EPR spectrum. At least two signals are observed in the g≈2 region, denoted (rhombic) $g_{1,2,3}$ = 2.039, 2.015 and 2.004 and (axial) $g_{\perp}$= 2.033, $g_{\parallel}$ = 2.027, as determined through simulations (Fig. S20). The amplitudes of both components show a maximum at 10 K and both diminish in a similar fashion at elevated temperatures (Fig. S18). The power dependence of the amplitudes of 1-3 (as indicated in Fig. S19, panel A) is shown in Fig. S19, panels B-D.

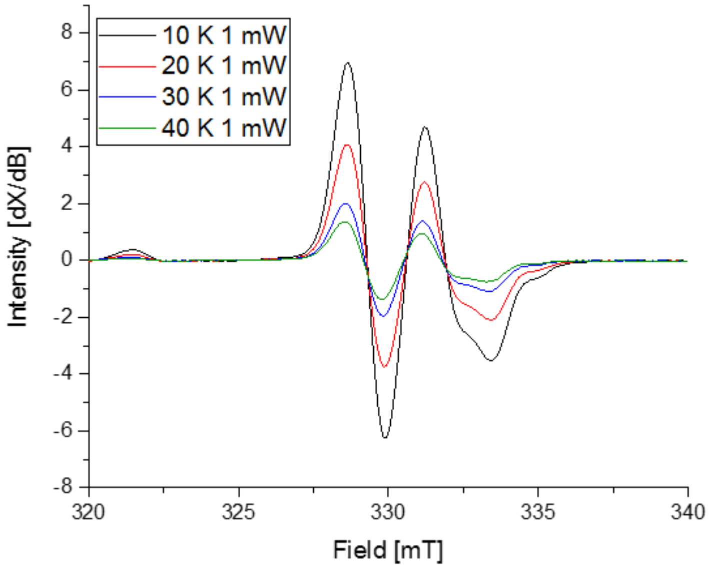

Figure S18. EPR spectra of 0.25 mM **4**⁻ treated with 0.25 mM CoCp* at – 40 ºC recorded at 1 mW, 10 K (black spectrum), 20 K (red spectrum), 30 K (blue spectrum) or 40 K (green spectrum).

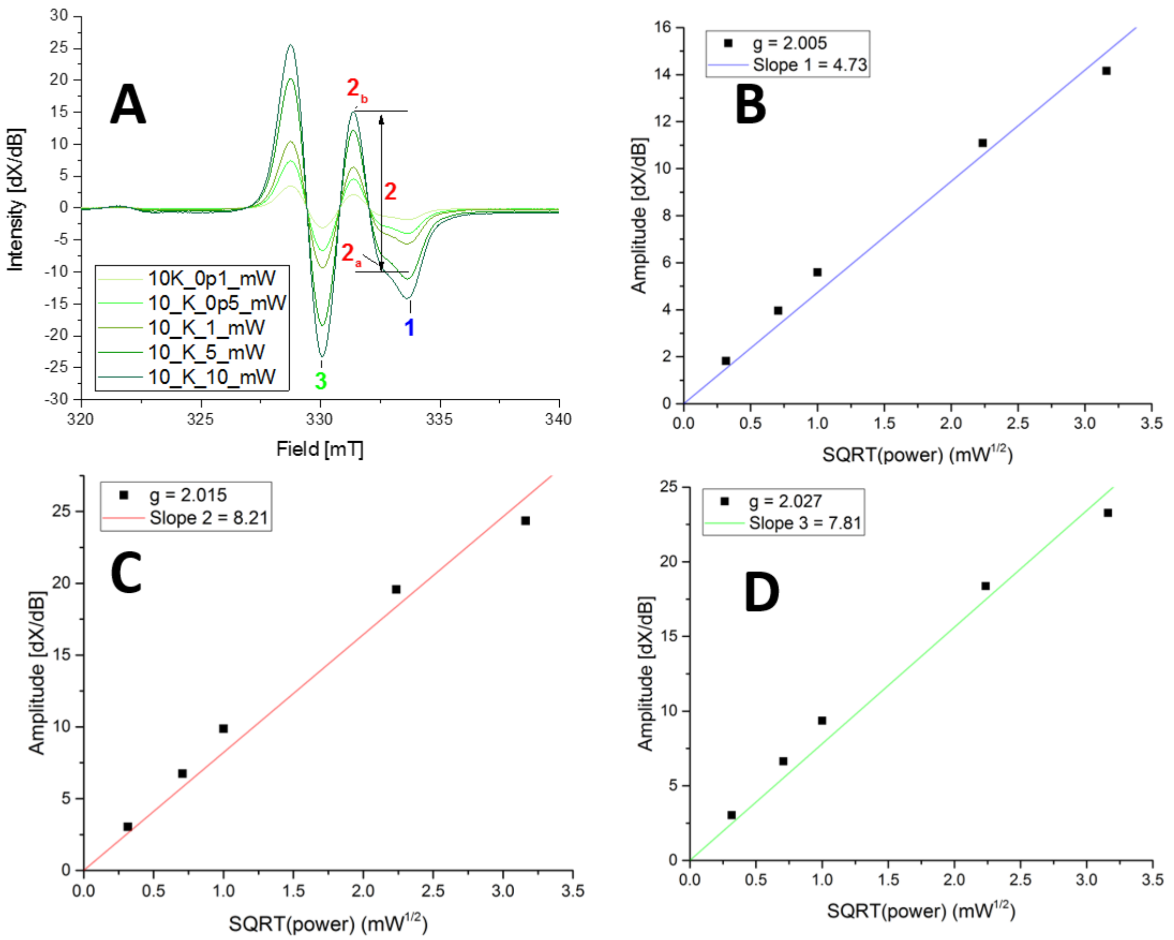

Figure S19. Panel A: EPR spectrum and power dependence of **4**⁻ + CoCp*. Recorded at 10 K, microwave power: 0.1 mW, 0.5 mW, 1 mW, 5 mW or 10 mW. Panels B-C: The signal amplitude when plotted against the square root of power at position 1 (B), position 2, peak to trough amplitude (C) and position 3 (D) demonstrate that the observed species do not saturate easily at the given temperature (10 K).

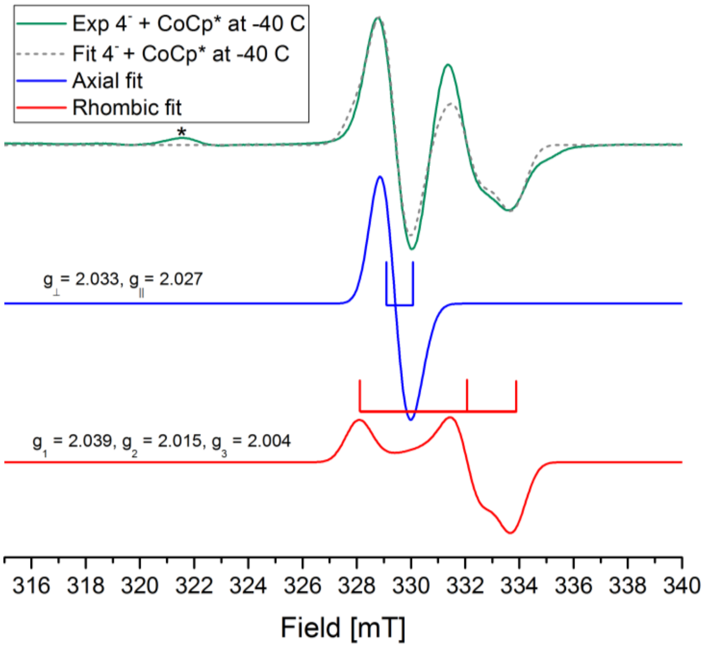

Figure S20. Simulations of **4**⁻ reduced at -40 °C using 1 eq CoCp*. Top: The green line shows the experimental spectrum, the asterisk indicates a small impurity, the dashed line is the simulated spectrum (from simulations using Easyspin[4, 5]) consisting of an axial signal with g-values of $g_\perp$= 2.033, $g_\parallel$ = 2.027, and a rhombic signal with apparent g-values of $g_{1,2,3}$ = 2.039, 2.015 and 2.004 (ratio 3:1 rhombic:axial signal intensities). Middle: The blue line shows the simulated axial signal. Bottom: The red line shows the simulated rhombic spectrum. g-values are marked by the bars.

SI_14 EPR control experiments

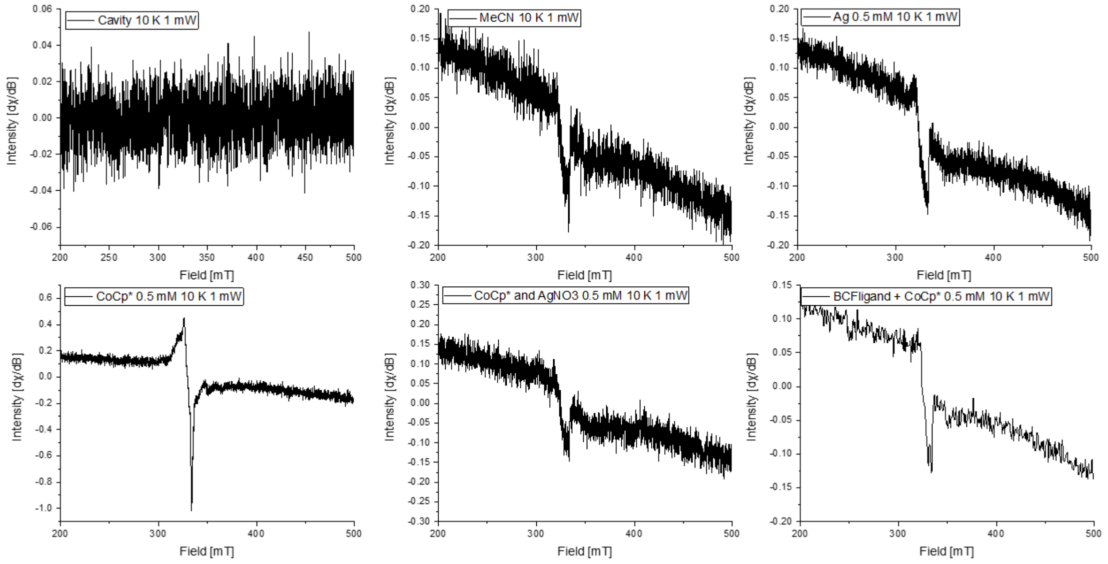

Figure S21. Selected EPR control experiments. Top left: EPR of the cavity, top middle: EPR of the solvent (acetonitrile), Top right: EPR of 0.5 mM $AgNO_3$. Bottom left: EPR of 0.5 mM CoCp*, bottom middle: EPR of 0.5 mM CoCp* and 0.5 mM $AgNO_3$ confirms that oxidised CoCp* is EPR silent and reduced $AgNO_3$ is also EPR silent, bottom right: EPR of 0.5 mM CoCp* and 0.5 mM tris(pentafluorophenyl)borane (BCF), demonstrates that the CoCp* does not form a free radical with BCF. Note the small EPR signal amplitudes in all panels.

SI_ 15 cyclic voltammetry of 3^{2-}, full titration with HCl

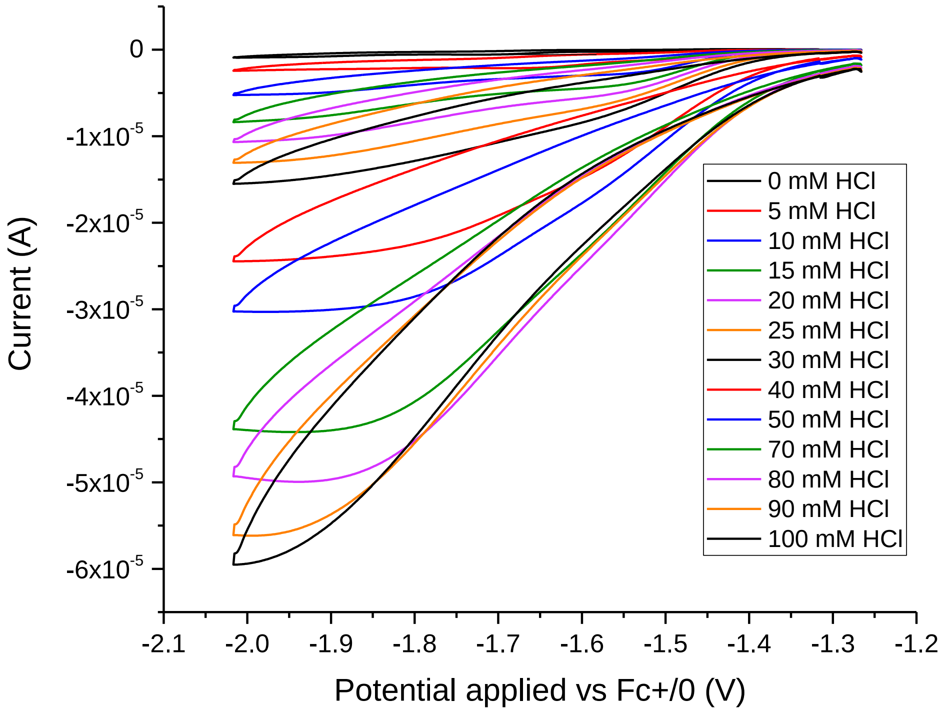

Figure S22. Cyclic voltammograms in the potential range -1.25 V to -2.01 V vs. Fc+/0 showing the effect of adding HCl to 5 mM $\mathbf{3}^{2-}$ on the current. This figure shows all titration points from 0-20 eq HCl (0 mM – 100 mM). Figure S23 confirms that these currents are a result of catalysis by showing negligible current when acid is added to the electrochemical cell in the absence of $\mathbf{3}^{2-}$.

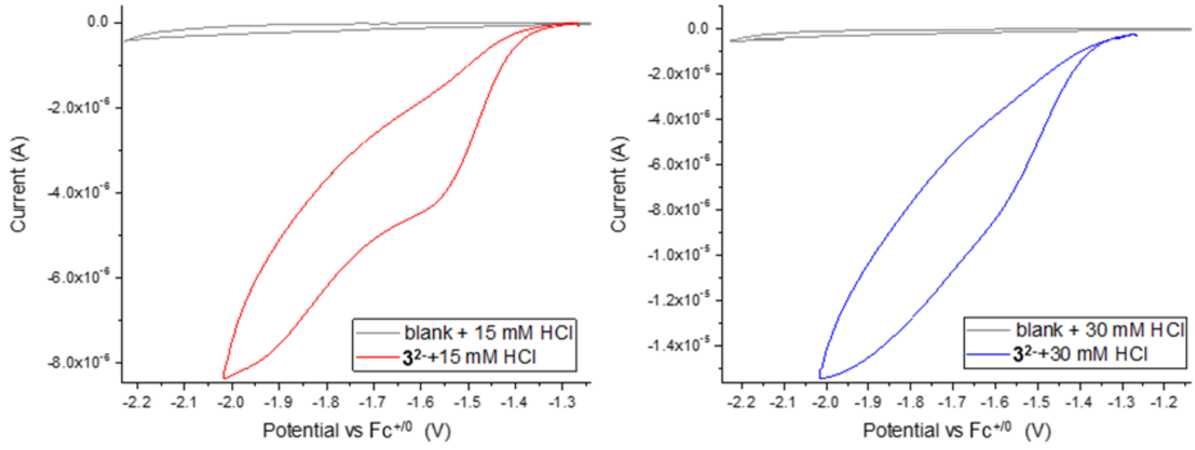

Figure S23. Cyclic voltammograms showing the trace of the blank electrochemical cell in the presence of acid and absence of $\mathbf{3}^{2-}$ as compared to the electrochemical cell containing 5 mM $\mathbf{3}^{2-}$ in the presence of acid (left, 15 mM HCl; right, 30 mM HCl). These control experiments demonstrate that the growth in current observed when acid is added to complex $\mathbf{3}^{2-}$ is a result of catalysis by $\mathbf{3}^{2-}$.

SI_16 Peak current plotted against concentration of HCl at -1.95 V and -1.55 V vs Fc+/0

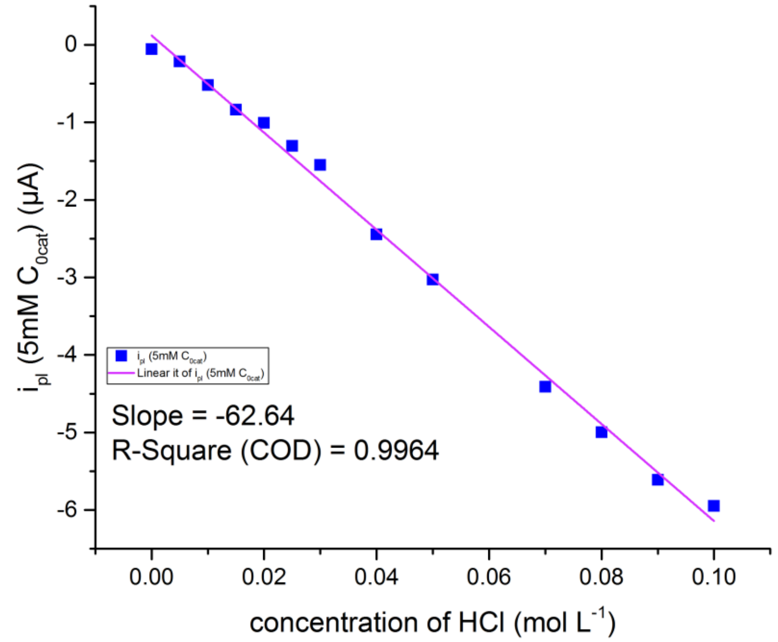

Figure S24. Plot of the peak current (i_{pl}) at -1.95 V vs. Fc $^{+/0}$ against the concentration of acid. The line shows a linear dependence of the current on the HCl concentration. No distinct plateau current is reached at this potential within the available titration range. [**3**$^{2-}$] = 5 mM, scan rate = 100 mV s^{-1}

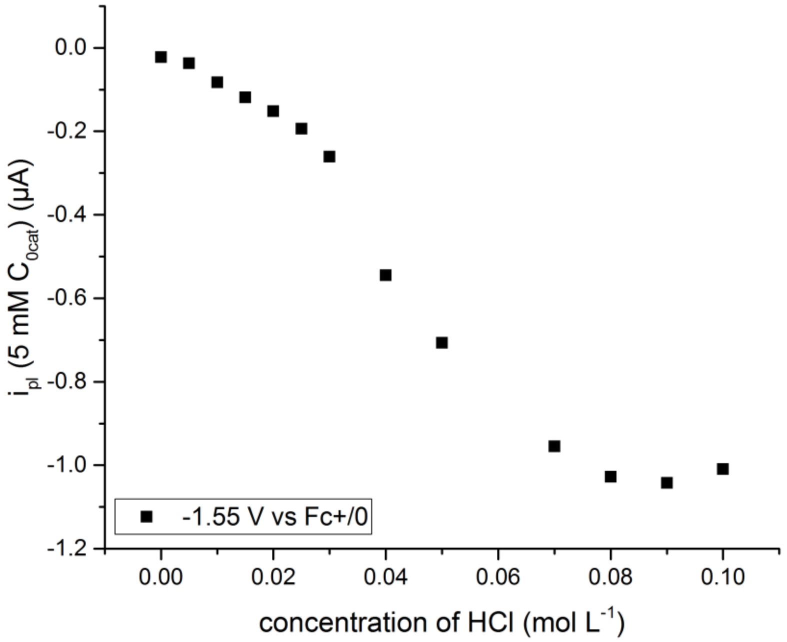

Figure S25 Plot of the peak current (i_{pl}) at -1.55 V vs Fc $^{+/0}$ against the concentration of acid. The line shows a non-linear dependence of the current on the HCl concentration at this potential. A plateau current is observed at ≥80 mM HCl. [**3**$^{2-}$] = 5 mM, scan rate = 100 mV s^{-1}.

SI_17 X-ray absorption spectroscopy

Table S4. EXAFS simulation parameters. R, Å; N, per Fe; $2\sigma^2$, Å^2. Data refer to EXAFS spectra in Figure 4 (main text). *Coordination numbers were fixed in the simulations, ms refers to a multiple-scattering shell, the given error sum, R_F, was calculated for 1-3 Å of reduced distance in the Fourier-transforms

shell	**Fe-C**	**Fe-O**	**Fe-C**	**Fe-N**	**Fe-S**	**Fe-Fe**	**Fe-C**	**Fe-C=N/O$_{ms}$**
**2^{2-}** in MeCN								
R	1.76	2.97	1.89	3.19	2.26	2.50	3.34	1.29
N	2*	2*	1*	1*	2*	1*	2*	3*
$2\sigma^2$ x10^3	5	1	1	2	6	7	2	9
R_F = 13.4 %								
**3^{2-}** in MeCN								
R	1.74	2.98	1.87	3.18	2.25	2.50	3.33	1.28
N	2*	2*	1*	1*	2*	1*	2*	3*
$2\sigma^2$ x10^3	6	2	1	1	4	3	9	4
R_F = 12.1 %								
**4^{-}** in MeCN								
R	1.78	3.00	1.87	3.26	2.26	2.54	3.30	1.22
N	2*	2*	1*	1*	2*	1*	2*	3*
$2\sigma^2$ x10^3	4	1	2	2	5	4	11	7
R_F = 6.7 %								
**5** in MeCN								
R	1.78	2.99	1.92	3.26	2.26	2.53	3.27	1.22
N	2*	2*	1*	1*	2*	1*	2*	3*
$2\sigma^2$ x10^3	1	1	2	1	7	6	2	5
R_F = 15.2 %								
**6** in MeCN								
R	1.79	3.11	-	-	2.23	2.48	3.37	0.97
N	3*	3*	-	-	2*	1*	2*	3*
$2\sigma^2$ x10^3	1	1	-	-	3	1	1	2
R_F = 12.4 %								

*Coordination numbers were fixed in the simulations, ms = multiple scattering, the error sum, R_F, was calculated for 1-3 Å of reduced distance.

.

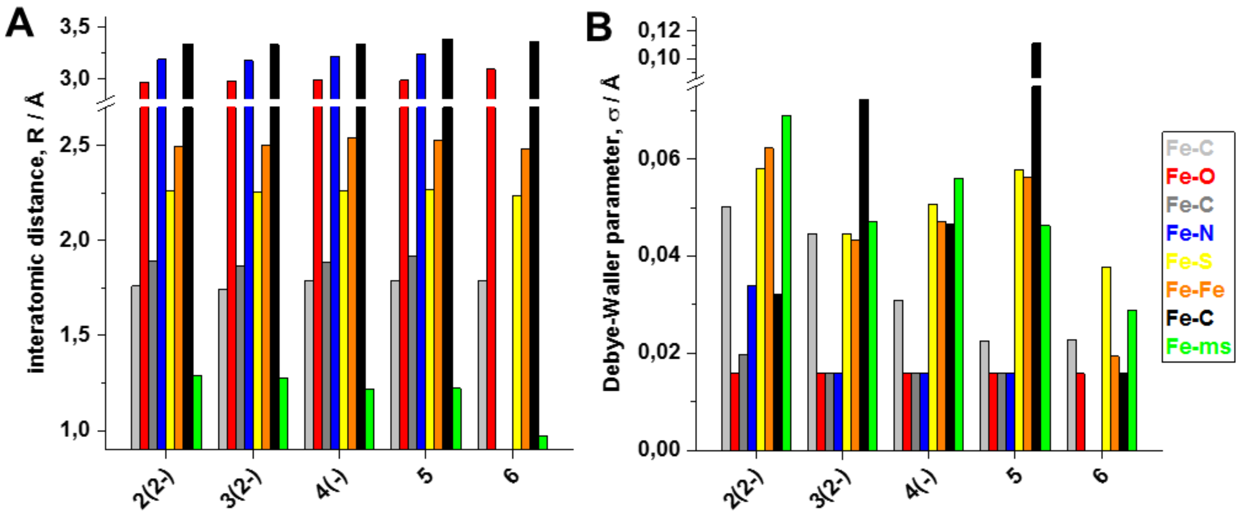

Figure S26. EXAFS simulation parameters. (A) interatomic distances, R (in Å). (B) Debye-Waller parameters, σ (in Å). Data corresponds to fit results in Table S4 (the legend on the right annotates respective interatomic interactions; ms, multiple scattering).

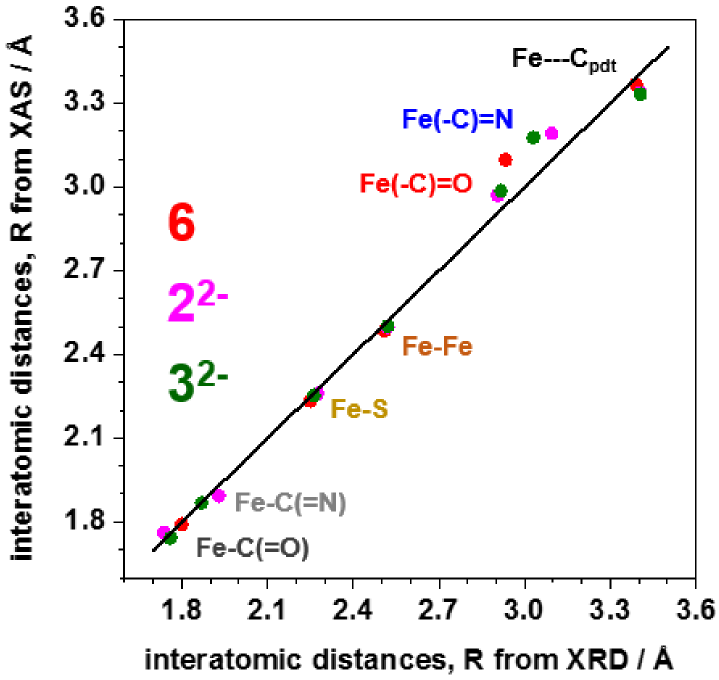

Figure S27. Comparison of interatomic distances from XAS and XRD. Symbols show results for selected Fe complexes from EXAFS analysis (solution data) and crystal structures (solid state data). The line shows the diagonal (ideal correlation).

SI_references

1. N. G. Connelly and W. E. Geiger, *Chemical Reviews*, 1996, **96**, 877-910.
2. E. S. Rountree, D. J. Martin, B. D. McCarthy and J. L. Dempsey, *ACS Catalysis*, 2016, **6**, 3326-3335.
3. B. C. Manor, M. R. Ringenberg and T. B. Rauchfuss, *Inorganic Chemistry*, 2014, **53**, 7241-7247.
4. S. Stoll, in *Multifrequency Electron Paramagnetic Resonance*, 2014, DOI: https://doi.org/10.1002/9783527672431.ch3, pp. 69-138.
5. S. Stoll and A. Schweiger, *Journal of Magnetic Resonance*, 2006, **178**, 42-55.
